# Survival Outcomes with IL-2-Free Dinutuximab Beta Treatment Strategies With or Without 13-cis-Retinoic Acid in Relapsed or Refractory Neuroblastoma: An Exploratory Unanchored, Population-Adjusted Comparison of Two Prospective Cohorts

**DOI:** 10.3390/biomedicines14102200

**Published:** 2026-09-29

**Authors:** Holger N. Lode, Przemysław Holko, Aleksandra Wieczorek, Katarzyna Śladowska, Nikolai Siebert, Dominique Valteau-Couanet, Alberto Garaventa, Adela Cañete, John Anderson, Isaac Yaniv, Shifra Ash, Lucas Moreno, Juliet Gray, Roberto Luksch, Genevieve Laureys, Cormac Owens, Carla Manzitti, Sascha Troschke-Meurer, Paweł Kawalec, Ruth L. Ladenstein

**Affiliations:** 1 Department of Pediatric Hematology and Oncology, University Medicine Greifswald, 17475 Greifswald, Germany; 2 Department of Nutrition and Drug Research, Institute of Public Health, Faculty of Health Sciences, Jagiellonian University Medical College, 31-008 Krakow, Poland; 3 Department of Pediatric Oncology and Hematology, Institute of Pediatrics, Jagiellonian University Medical College, 31-008 Krakow, Poland; 4 Children and Adolescent Oncology Department, Gustave Roussy, Paris-Sud University, 94805 Paris, France; 5 Oncology Unit, IRCCS Istituto Giannina Gaslini, 16147 Genoa, Italy; 6 Hospital Universitario y Politecnico La Fe, 46026 Valencia, Spain; 7 UCL Great Ormond Street Institute of Child Health, London WC1N 1EH, UK; 8 Schneider Children’s Medical Center of Israel, Petach Tikva 4920235, Israel; 9 Adelson School of Medicine, Ariel University, Ariel 4070000, Israel; 10 Division of Pediatric Hematology & Oncology, Vall d’Hebron Barcelona Hospital Campus, Passeig Vall d’Hebron 119, 08035 Barcelona, Spain; 11 Centre for Cancer Immunology, University of Southampton, Southampton SO16 6YD, UK; juliet.gray@uhs.nhs.uk; 12 Pediatric Oncology Unit, Fondazione IRCCS Istituto Nazionale dei Tumori, 20133 Milan, Italy; 13 Department of Pediatric Hematology/Oncology and Stem Cell Transplantation, Ghent University Hospital, 9000 Ghent, Belgium; genevieve.laureys@ugent.be; 14 Department of Haematology and Oncology, Children’s Health Ireland, D12 N512 Dublin, Ireland; 15 Centrum HTA Sp. z o.o., 31-232 Kraków, Poland; 16 St. Anna Children’s Hospital, St. Anna Children’s Cancer Research Institute and Department of Pediatrics, Medical University Vienna, 1090 Vienna, Austria

**Keywords:** neuroblastoma, dinutuximab beta, isotretinoin, 13-cis-retinoic acid, event-free survival, overall survival, propensity score, landmark analysis

## Abstract

**Background/Objectives**: Survival outcomes with IL-2-free dinutuximab beta (DB) treatment strategies with and without 13-cis-retinoic acid (RA) have not been directly compared in a randomized study. We compared event-free survival (EFS) and overall survival (OS) between two study-defined cohorts with relapsed or refractory neuroblastoma receiving DB with or without RA. **Methods**: We retrospectively reanalyzed individual patient data from APN311-304 study (DB without RA, *n* = 36) and the experimental arm of APN311-202 V3 study (DB plus RA, *n* = 44). Eligibility and time zero were harmonized, and overlap-weighted Kaplan–Meier and Cox analyses were performed. An exploratory post-end-of-treatment landmark analysis was conducted among patients who remained alive and EFS-event-free through the landmark. **Results**: In the primary overlap-weighted analysis, no clear difference was detected between the cohorts for event-free survival (EFS; hazard ratio [HR], 0.92; 95% confidence interval [CI], 0.31–2.73) or overall survival (OS; HR, 1.95; 95% CI, 0.46–8.20). Cohort-specific effective sample sizes were 10.15 for no RA and 15.30 for RA versus nominal sizes of 36 and 44, respectively. Exploratory conditional landmark estimates were also imprecise for EFS (HR, 1.24; 95% CI, 0.37–4.12) and OS (HR, 0.44; 95% CI, 0.07–2.71). **Conclusions**: This exploratory comparison did not provide evidence of more favorable EFS or OS in the RA cohort than in the no-RA cohort. However, the estimates were highly imprecise and compatible with clinically important benefit or harm. The findings should not be interpreted as evidence of equivalence or as evidence that RA provides no benefit and should not alone guide continuation or omission of RA. Because RA use was perfectly aligned with the source study, the comparison cannot isolate a causal RA effect.

## 1. Introduction

Dinutuximab beta (DB; Qarziba^®^) is a chimeric monoclonal antibody directed against disialoganglioside 2 (GD2), a cell surface antigen that is highly expressed on neuroblastoma cells but has limited expression in normal tissues. By binding to GD2, DB mediates tumor cell destruction primarily through immune effector mechanisms, including antibody-dependent cellular cytotoxicity and complement-dependent cytotoxicity, thereby enhancing the host antitumor immune response [1,2,3,4,5]. The rationale for targeting GD2 in neuroblastoma is well established, and anti-GD2 antibodies have become a cornerstone of immunotherapeutic strategies for this disease [1,5].

In Europe, DB is an established component of maintenance immunotherapy for patients with high-risk neuroblastoma and is also indicated for patients with a history of relapsed or refractory disease after stabilization of active disease [4]. In contemporary protocols, DB has been administered alone or with other agents, including interleukin-2 (IL-2) and 13-cis-retinoic acid (isotretinoin, RA). Randomized evidence showed that adding subcutaneous IL-2 to DB and RA increased toxicity without improving event-free survival, supporting IL-2-free DB regimens [6]. Whether EFS or OS differs between IL-2-free DB treatment strategies with and without RA remains unresolved.

RA is a differentiation agent rather than a conventional cytotoxic drug. In neuroblastoma models, 13-cis-RA and its active metabolites promote neurite outgrowth, inhibit proliferation, and can reduce MYCN expression through retinoid-receptor signaling [7]. These effects provide a biological rationale for using RA in a minimal-residual-disease setting, in which differentiation or growth arrest of residual tumor cells may complement immune-mediated tumor-cell killing by DB. The clinical precedent derives largely from post-consolidation treatment in newly diagnosed high-risk neuroblastoma, where earlier follow-up of CCG-3891 favored RA over no further therapy, although the difference was attenuated with longer follow-up [8,9]. These data do not isolate the contribution of RA when an anti-GD2 antibody is already present.

A more specific interaction with anti-GD2 therapy is biologically plausible but incompletely established. In preclinical experiments, all-trans retinoic acid maintained GD2 expression in most tested neuroblastoma cell lines and enhanced antibody-dependent cellular cytotoxicity mediated by the anti-GD2 antibody Hu14.18K322A [10]. However, those experiments used all-trans retinoic acid, a different anti-GD2 antibody, and short-term in vitro or ex vivo systems. They therefore support a testable hypothesis of complementarity, not clinical proof of synergy between isotretinoin and DB. No randomized component trial has directly compared DB plus RA with DB alone in a clinically comparable R/R (relapsed or refractory) population. More broadly, patient advocates have emphasized the need to critically reassess components of intensive multimodal neuroblastoma therapy that may increase treatment burden [11].

For R/R neuroblastoma, prospective phase II data have shown that single-agent DB administered as a long-term infusion is active and well tolerated, with clinically meaningful response rates and 3-year progression-free survival (PFS) and overall survival (OS) rates of 31% and 66%, respectively [2]. Broader reviews emphasize that the treatment of high-risk and relapsed neuroblastoma is multimodal and that further therapeutic advances remain necessary [3].

The specific contribution of RA to clinical outcomes when coadministered with DB in relapsed or refractory neuroblastoma remains unclear. No randomized study has directly compared DB plus RA with DB alone in a clinically comparable maintenance population, and the available evidence was not designed to isolate the independent effect of RA in this setting [3]. Addressing this question using conventional observational designs or indirect comparisons is methodologically challenging because treatment exposure accumulates over time, whereas early relapse, disease progression, or death may shorten treatment duration. Consequently, naïve analyses that do not account for the timing of treatment exposure or cumulative dose are susceptible to guarantee-time or immortal-time bias. Landmark analyses, time-dependent exposure definitions, and inverse probability weighting are among the recommended approaches when treatment assignment or classification depends on post-baseline events [12,13,14,15,16,17,18].

We therefore performed an exploratory, hypothesis-generating comparison of individual patient data from two prospective cohorts treated with IL-2-free DB strategies with or without RA. After harmonizing eligibility criteria and time zero, we used propensity-score overlap weighting to compare EFS and OS between the study-defined cohorts in the population represented by patients with overlapping measured baseline characteristics. Because RA use was perfectly aligned with the source study, any observed between-cohort differences could not be attributed independently to RA rather than to unmeasured patient-level or protocol-level differences. The post-EOT (post-end-of-treatment) landmark analysis was exploratory and conditional on remaining alive and EFS-event-free through the landmark.

## 2. Materials and Methods

### 2.1. Study Design

This was an exploratory unanchored indirect comparison of time-to-event outcomes using IPD (individual patient data) from prospective studies evaluating DB in patients with R/R neuroblastoma. Both unadjusted (naïve) and population-adjusted analyses were performed, with the latter using inverse probability weighting. Because treatment strategy was completely determined by the source study, the analysis could reduce measured patient-level imbalance but could not separate an RA effect from unmeasured patient-level or protocol-level differences. The validity of the comparison depended on the assumption that all prognostic factors and treatment-effect modifiers that differed between studies were measured and adequately controlled through eligibility restriction and weighting.

The following two cohorts were included in the comparison:

- Non-RA cohort comprising patients with R/R neuroblastoma who received DB monotherapy, without concomitant RA or IL-2; and
- RA cohort comprising patients with R/R neuroblastoma who received DB in combination with RA, without concomitant IL-2.

Harmonized inclusion criteria were applied across both study cohorts. To ensure analytical consistency, a common time zero for EFS and OS was defined. In the base-case analysis, time zero for both EFS and OS was the date of the first DB administration in each cohort. This alignment was intended to improve comparability by using a common index date; it does not account for differences in supportive care, eligibility, assessment schedules, or other study-level factors. We sought to address these limitations by applying harmonized eligibility criteria across the two study cohorts and using adjusted indirect comparison methods based on propensity-score (PS) weighting to balance the cohorts with respect to key baseline prognostic factors.

Recent findings indicate that greater DB exposure was associated with both EFS and OS [19]. The effects of RA and DB may be interdependent, and exposure to both treatments accumulates over time. Events such as disease progression, relapse, or death (which define EFS or OS) frequently terminate or shorten treatment, such that patients with early progression typically receive fewer treatment cycles and a lower cumulative DB dose. Consequently, simply adjusting for DB exposure when comparing the RA and no-RA cohorts may introduce substantial bias, including immortal-time bias (where patients must survive long enough to receive the full planned treatment) and reverse causation (where a lower cumulative DB dose is a consequence, rather than a cause, of shorter EFS or OS) [14,15]. Therefore, in a complementary analysis, post-treatment EFS and OS were evaluated only among patients who remained alive and EFS-event-free through a post-EOT landmark defined as 25 days after the last DB administration. Weighted landmark analyses were then performed conditional on reaching this landmark.

Conditioning on survival to the post-EOT landmark may itself introduce selection bias. Therefore, stabilized inverse probability of selection weights (IPSWs) for inclusion at the post-EOT landmark were estimated using baseline and pre-landmark covariate history [16,17]. The weights were then truncated at the 1st to 99th percentiles to reduce variance inflation [18]. Finally, weighted survival models were fitted from the post-EOT landmark to compare post-EOT EFS and OS between the RA and no-RA cohorts. The post-EOT landmark analysis was exploratory and conditional on patients remaining alive and EFS-event-free through the landmark. It therefore describes a selected post-treatment risk set and should not be interpreted as an intention-to-treat comparison or as confirmation of the base-case analysis.

### 2.2. Data Sources and Patient Selection

Among the prospective and protocol-defined DB cohorts considered for this assessment (Table 1), APN311-304 [2] was the only prospective source that provided individual patient data on DB monotherapy (i.e., without IL-2 or RA). APN311-202 V3 [20] provided the closest eligible source for DB plus RA without IL-2 that met the prespecified criteria. To compare long-term outcomes between patients receiving IL-2-free DB with and without RA, it was necessary to identify a data source that was as clinically comparable as possible to APN311-304 and that included DB in combination with RA.

The selection criteria were as follows: (i) patients with R/R neuroblastoma after stabilization of active disease, consistent with the disease setting in the DB indication [4]; (ii) an intended DB schedule matching the target regimen (up to five 35-day cycles, with DB administered over 5 or 10 days per cycle); (iii) no concomitant IL-2 with DB; (iv) an intended RA schedule consistent with clinical guidelines/practise (v) prospective study design; and (vi) availability of IPD.

The experimental arm of the APN311-202 V3 (stage II) study [20] met the predefined inclusion criteria.

Several differences were identified between the APN311-304 and APN311-202 V3 studies with respect to the inclusion and exclusion criteria, study design, and planned treatment regimens (Supplementary Table S2). APN311-202 V3 enrolled patients with HR-NBL following high-dose chemotherapy and stem cell rescue, whereas APN311-304 included patients with R/R disease that had been controlled with conventional therapy and also permitted enrollment of patients with early minimal bone marrow relapse (up to nine patients in APN311-304 had not received first-line high-dose therapy; information on subsequent stem cell transplantation in these patients was unavailable). The eligibility criteria were otherwise broadly comparable with respect to key indicators of clinical stability, including the absence of progressive disease at study entry, a performance status ≥ 70, a life expectancy of ≥12 weeks, a washout period of ≥2 weeks, and similar cardiopulmonary and laboratory eligibility requirements. However, APN311-202 V3 excluded all patients with prior exposure to DB, whereas APN311-304 excluded only patients who were anti-drug antibody–positive following previous anti-GD2 therapy (affected at least four patients in APN311-304).

Explicit alignment of time zero was required because the APN311-202 V3 study used randomization as the primary starting point for EFS, while the APN311-304 study was a single-arm study in which OS and PFS were secondary endpoints. Therefore, the indirect comparison used a harmonized time zero (e.g., the first administration of DB or an EOT landmark), consistent with the prespecified analytical approach. In addition, eligibility in the APN311-304 study was restricted to patients with evidence of disease at baseline, whereas the APN311-202 V3 study also allowed enrollment of patients without evidence of disease. This eligibility difference could have favored APN311-202 V3 because absence of detectable disease is prognostically favorable. To improve comparability with the APN311-304 study, patients in APN311-202 V3 without evidence of disease at baseline were excluded from the indirect comparison (i.e., patients without disease measurable by magnetic resonance imaging and/or computed tomography, and without disease evaluable by metaiodobenzylguanidine [MIBG] scintigraphy, technetium scintigraphy, or bone marrow histology). The harmonized inclusion and exclusion criteria for the indirect comparison are presented in Table 2. In simple terms, the RA cohort was drawn from APN311-202 V3, a randomized trial in which patients had undergone high-dose chemotherapy and stem-cell rescue, whereas the no-RA cohort was drawn from APN311-304, a single-arm relapsed/refractory study in which active disease had been stabilized with conventional treatment. The studies also differed in prior anti-GD2 exposure, eligibility regarding detectable disease, assessment schedules, calendar period, and subsequent therapy. Eligibility restriction, use of a common time zero, and weighting could address measured patient-level differences only; they could not eliminate differences inherent to the source studies.

Applying these criteria resulted in the inclusion of 80 patients: 36 from the APN311-304 study (DB without RA) and 44 from the APN311-202 V3 study (DB plus RA) (Figure 1).

### 2.3. Data Management

The present manuscript reports a secondary analysis and was not separately registered as a clinical trial. The original studies were registered as follows: APN311-304, ClinicalTrials.gov NCT02743429 and EudraCT 2014-000588-42; and APN311-202, ClinicalTrials.gov NCT01701479 and EudraCT 2009-018077-31.

This study was a retrospective reanalysis of IPD from these two prospective clinical trials. Eligible patients were selected from the full analysis set (FAS) of the APN311-304 study and from the experimental arm of the APN311-202 V3 study (DB plus RA, without IL-2) within its FAS (Figure 1). The following information was extracted for each patient:

#### 2.3.1. Patient Characteristics, Determined According to Availability in Both Cohorts

- date of birth, for patients from APN311-304, only the year of birth was available; therefore, a birth date of June 30 was assumed for each patient; this variable was used to calculate age at study entry and age at initial diagnosis;
- sex, categorized as male or female;
- date of initial diagnosis, used to calculate time from diagnosis in months and calendar year of diagnosis;
- date of immunotherapy initiation, defined as the first administration of DB;
- date of DB treatment completion, defined as the date of the last DB administration; this date was subsequently used to derive the post-EOT landmark by adding 25 days. For patients in APN311-202 V3 with a missing date of the last DB administration, the date was estimated from the number of treatment cycles, the 35-day cycle length, and the date of the first DB administration;
- *MYCN* status (amplified, non-amplified, or missing);
- International Neuroblastoma Staging System (INSS) stage at initial diagnosis (INSS stage or missing);
- frontline high-dose therapy (busulfan and melphalan [BuMEL]; carboplatin, etoposide, melphalan [CEM], or MIBG therapy);
- frontline treatment (local therapy, maintenance therapy, radiotherapy, surgery, and anti-GD2 maintenance therapy);
- baseline performance status (Lansky or Karnofsky score);
- baseline MIBG scan findings (soft tissue, distant lymph nodes, liver, and skeletal lesions);
- number of DB treatment cycles, DB dose per cycle, and cumulative DB dose per m^2^;
- number of RA treatment cycles and cumulative RA dose (mg).

#### 2.3.2. Outcomes

- date of censoring (date of the last follow-up assessment or last contact);
- date of death;
- date of the EFS event, defined as relapse, disease progression, other malignancy, or death;
- OS event indicator (event, 1; no event—censored, 0);
- EFS event indicator (event, 1; no event—censored, 0);

The following additional modifications were made to the database: (i) correction of a dosage entry error (decimal point error) for one patient in the APN311-202 V3 study; and (ii) recoding of a baseline performance status value of 0 as missing for one patient in the APN311-202 V3 study because it was considered a probable data entry error.

### 2.4. RA Treatment

In the APN311-202 V3 study, RA was administered at a dose of 160 mg/m^2^/day or, for patients weighing > 12 kg who were unable to swallow capsules, 200 mg/m^2^/day [24]. It was administered twice daily for 14 days of each treatment cycle, starting after completion of the DB infusion.

The study collected data on the number of RA cycles initiated and the cumulative RA dose administered across all cycles (expressed as milligrams per patient). The number of cycles initiated was designated the primary descriptive RA exposure metric; this choice parallels the use of initiated cycles for DB exposure [19].

### 2.5. Statistical Analysis

#### 2.5.1. Endpoints

The study included two efficacy endpoints:

- EFS: time to the first occurrence of disease progression, relapse, secondary neoplasm, or death from any cause;
- OS: time to death from any cause.

The endpoints used in the present analysis were consistent with those of the original studies, except for APN311-304, in which PFS was the original endpoint (Supplementary Table S3).

For APN311-304, the harmonized time-to-event endpoint was derived from the available information on progression, relapse, and death. Information on secondary neoplasms was not available in the extracted dataset. Consequently, ascertainment of the secondary-neoplasm component of EFS could not be fully harmonized between the source studies.

The target contrast was between two trial-defined treatment strategies rather than between RA and no RA under otherwise identical conditions. Because treatment strategy was completely determined by the source study, the validity of the comparison required the strong assumption that all prognostic factors and treatment-effect modifiers differing between studies were measured, harmonized, and adequately controlled. Study-level effects could not be separated from RA use.

The original time zero for EFS and OS (baseline, study entry, or randomization) was harmonized across both cohorts. In the base-case analysis, time zero for EFS and OS was defined as the date of the first DB administration in both cohorts. In additional analyses evaluating post-treatment survival, time zero for EFS and OS was set at the post-EOT landmark. The post-EOT landmark was defined as the date of the last DB administration plus the standard 25-day interval between consecutive DB administrations used in the long-term infusion schedule. The 25-day interval was selected to avoid classifying patients as post-treatment while they were still within the expected interval before the next planned DB cycle. Patients who experienced an event before or on the post-EOT landmark were excluded from the analyses [19].

Safety endpoints were not analyzed because they were not comparable between the two studies. The methods used to collect and grade adverse events differed between study protocols and reflected differences in the periods during which the studies were conducted. Furthermore, RA-related adverse events (e.g., dyslipidemia, hepatic abnormalities, and mucocutaneous toxicities) are influenced by the intensity of safety monitoring, further limiting the validity of direct comparisons.

#### 2.5.2. Covariates

The following covariates, considered potential predictors of clinical outcomes, were available and were considered in the descriptive and/or adjusted analyses: age at study entry (years); time from initial diagnosis to study entry (months); calendar year of diagnosis; country of residence; INSS stage at diagnosis (stage 4 vs. other); *MYCN* status at diagnosis (amplified vs. other); sex (male or female); frontline high-dose therapy (BuMEL, CEM, or MIBG therapy); frontline treatment (local therapy, maintenance therapy, radiotherapy, surgery, and anti-GD2 maintenance therapy); baseline performance status (Lansky or Karnofsky score); baseline MIBG scan findings (soft tissue, distant lymph nodes, liver, and skeletal lesions); and DB exposure, including the number of treatment cycles initiated, the number of cycles with a dose of at least 50 mg/m^2^, and the cumulative DB dose (mg/m^2^).

Baseline laboratory prognostic markers (e.g., ferritin and lactate dehydrogenase [LDH]) and baseline response status following re-induction/stabilization therapy were excluded from the analyses because the data were unavailable or incomplete. In the APN311-304 study, ferritin measurements and LDH measurements were missing for 37 and 35 patients, respectively, and no information on response status before study enrollment was available. Bone marrow assessment was also excluded because the data were unavailable in the APN311-202 V3 study.

#### 2.5.3. Primary Handling of Missing Data

Patients with multiple missing characteristics or outcomes were planned to be excluded from the base-case analysis (there were only 2 exclusions from available full-analysis set (Figure 1). There was no missing data adjustment for continuous variables (no missing continuous data). The categorical variables (MYCN, performance scale) were extended with an explicit “Missing” category as an additional variable level. However, only one patient from no-RA cohort had missing MYCN data, hence the patient was included in the analysis under cumulative category level with non-amplified MYCN (‘Other MYCN’). Although protocol eligibility required a performance status score of at least 70, the numerical baseline score was unavailable in the analytical dataset for 15/44 patients (34.1%) in the RA cohort but was available for all 36 patients in the no-RA cohort. Because this missingness differed systematically by source study, baseline performance status was excluded from the propensity-score model and was not used to assess or claim post-weighting balance. No deterministic assignment, complete-case restriction, or model-based imputation of baseline performance status was performed. Because all missing performance-status values occurred in one source-study cohort and the available data did not support a credible reconstruction of their distribution, these approaches would have required strong and unverifiable assumptions and would not have resolved source-study confounding. Candidate covariates that caused collinearity, complete or quasi-complete separation, or nonconvergence of the propensity-score model were prespecified for exclusion. However, no candidate covariate otherwise eligible for the base-case model met these criteria; therefore, no additional covariates were excluded for collinearity, separation, or nonconvergence.

#### 2.5.4. Comparison of Patient Characteristics

Baseline patient characteristics were compared between the cohorts using the χ^2^ test for categorical variables and the Wilcoxon rank-sum (Mann–Whitney U) test for continuous variables. For comparisons of weighted cohorts, continuous variables were compared using a test of the linear regression coefficient for the cohort indicator.

#### 2.5.5. PS Weighting

A population-adjusted comparison of EFS and OS was performed using propensity-score overlap weighting. Propensity score weighting was used to balance the treatment cohorts with respect to key baseline prognostic factors. Patient characteristics were included in the PS model if they met the following criteria: (i) availability in both studies; and (ii) sufficient frequency in each study cohort. Variables represented by fewer than 3 patients in either study were excluded from the base-case analysis to avoid assigning excessive weights to individual patients. In the base-case analysis, the following baseline characteristics were included in the adjustment process: age at DB initiation, time from initial diagnosis (months), sex, presence of soft tissue lesions on baseline MIBG scan, presence of skeletal lesions on baseline MIBG scan, INSS stage at diagnosis, disease status (relapsed or refractory), *MYCN* status, and type of frontline treatment (maintenance therapy; radiotherapy; surgery; high-dose MIBG therapy; high-dose BuMEL therapy; and rapid cisplatin, vincristine, carboplatin, etoposide, cyclophosphamide chemotherapy; Supplementary Table S4).

The two-level cohort variable (DB without RA vs. DB plus RA) was modeled using logistic regression with covariates measured at the initiation of DB treatment. To mitigate limited covariate overlap and reduce sensitivity to extreme inverse probability weights, overlap weighting was applied, targeting the covariate distribution of patients within the region of common support (i.e., the average treatment effect in the overlap population) [18,19,25].

Model selection and assessment were based on the Box–Tidwell test for linearity of continuous covariates in the logit, the generalized Hosmer–Lemeshow test, and the log-likelihood. Interactions between covariates and quadratic terms for continuous covariates were considered to accommodate nonadditivity and nonlinearity, respectively. Candidate terms were evaluated using likelihood-ratio (LR) tests comparing nested models and were retained when they improved model fit (two-sided α = 0.05). Terms were added using a hierarchy-preserving forward-selection procedure. Among candidate terms with *p* < 0.05, the term yielding the largest increase in the LR χ^2^ statistic was entered first. The model was then refitted, and the remaining candidate terms were re-evaluated in descending order of LR χ^2^. A newly added term was retained only if it continued to improve model fit (LR test *p* < 0.05); otherwise, it was removed.

Post-weighting covariate distributions were summarized descriptively using the available validated weighted outputs. The cohort-specific effective sample size was reported as a diagnostic measure [18,19,26]. *p* values for weighted baseline comparisons were retained as descriptive outputs only and were not interpreted as evidence of covariate balance or absence of imbalance.

Patients without an event were censored at the date of their last available assessment or at the applicable source-study data cutoff.

The weighted IPD were analyzed using a weighted Cox proportional hazards model with robust variance estimation and the weighted Kaplan–Meier method. Hazard ratios were parameterized for RA versus no RA; therefore, HR < 1 indicates a lower event hazard in the RA cohort. Effect estimates and 95% confidence intervals were the primary basis for interpretation. *p* values are reported descriptively and were not used to infer equivalence or the absence of a between-cohort difference. All analyses were performed using StataNow 19.5SE (StataCorp., College Station, TX, USA).

#### 2.5.6. Sensitivity Analyses

Several sensitivity analyses were prespecified, including a naïve comparison and post-landmark analyses with adjustment for DB exposure. The post-landmark analysis adjusted for DB exposure followed the methodology described previously [19]. Comparative survival analyses of the RA and no-RA groups were performed using a two-stage weighting approach to reduce bias arising from (i) selection into the post-landmark risk set and (ii) confounding in treatment assignment among patients who survived to the landmark.

First, the landmark analysis cohort was defined as patients who survived beyond the landmark without experiencing the EFS event, with follow-up commencing at the landmark. Stabilized IPSWs were estimated using a logistic regression model that included the cohort indicator and prespecified baseline and treatment covariates.

Among patients included in the landmark cohort, the PS for receiving RA was estimated using a logistic regression model that included the same covariates (excluding the treatment indicator) and DB exposure accumulated up to the landmark, defined as the number of DB cycles initiated [19]. Models were fitted with and without IPSWs. Propensity score overlap weights were then derived to target the population with clinical equipoise, with weights equal to 1 minus the PS for patients receiving RA and PS for patients not receiving RA.

Depending on the scenario, the final analysis weight was defined as (i) the product of the IPSWs and PS overlap weights, with both weights calculated independently; (ii) PS overlap weights estimated from a weighted PS model that incorporated IPSWs; (iii) the product of IPSWs and PS overlap weights, with the IPSWs estimated without including the cohort indicator; or (iv) PS overlap weights estimated from an unweighted PS model, targeting the population restricted to patients who were event-free at the EOT.

The sensitivity analysis scenarios are described in Supplementary Table S5.

No additional sensitivity analyses, missing-data reconstructions, subgroup analyses, or model variants were performed during the present revision.

## 3. Results

### 3.1. Patient Characteristics and Treatments

Before propensity-score weighting, notable differences were observed between the cohorts, particularly with respect to frontline treatment and baseline performance status (Supplementary Table S1). The cohorts also differed in subsequent treatment patterns; because these post-baseline treatments may reflect disease course, protocol differences, and clinical practice, they were not interpreted as measures of baseline comparability.

After weighting, the distributions of most covariates included in the propensity-score model became more similar between cohorts. Residual imbalance remained, particularly for five-cycle MSK m.N7 chemotherapy (*p* < 0.001). Baseline performance status was excluded from the propensity-score model because of study-dependent missingness and was found to vary between reweighted cohorts (*p* = 0.037). The statistically significant difference between cohorts in PS score was driven by missing data: *p* = 0.001 when the missing category was included in the unweighted comparison and *p* = 0.198 when the missing category was excluded The cohorts also differed in subsequent treatment patterns.

### 3.2. RA Exposure

In the APN311-202 V3 study, RA exposure data were available for all 44 patients with measurable or evaluable disease at baseline. Most patients (31/44, 70.5%) initiated five RA cycles, whereas fewer patients initiated two cycles (6/44, 13.6%), one cycle (3/44, 6.8%), four cycles (3/44, 6.8%), or three cycles (1/44, 2.3%). The mean number of cycles initiated was 4.20 (SD, 1.37; range, 1–5). The mean cumulative RA dose was 7237 mg (SD 3527), with a median of 7070 mg (interquartile range [IQR], 5600–8960) and a range of 700 to 21,000 mg. Among patients who initiated five cycles, the median cumulative dose was 8400 mg (IQR 7000–9240).

### 3.3. EFS and OS from the First DB Administration: PS-Weighted Analysis

In the overlap-weighted analysis from the first DB administration, no clear difference was detected between the two study-defined treatment cohorts for EFS (RA versus no RA: HR, 0.92; 95% CI, 0.31–2.73; *p* = 0.884) or OS (HR, 1.95; 95% CI, 0.46–8.20; *p* = 0.364; Figure 2). Both confidence intervals were wide and compatible with clinically important benefit or harm. The cohort-specific ESSs were 10.15 in the no-RA cohort and 15.30 in the RA cohort, compared with nominal sizes of 36 and 44, respectively. Because RA use was perfectly aligned with the source study, the estimates compared the two study-defined strategies rather than the independent causal effect of RA. In simple terms, patients whose baseline profiles were poorly represented in the other study received little weight, so the weighted survival contrasts were informed by a much smaller effective amount of data. This loss of information contributed to the wide confidence intervals and limited the ability to detect or rule out clinically relevant differences.

### 3.4. Sensitivity Analyses

#### 3.4.1. Naïve Comparison

In the unadjusted (naïve) analysis, the estimated EFS event hazard was lower in the RA cohort, but the confidence interval included the null (HR, 0.57; 95% CI, 0.31–1.04; *p* = 0.067). The OS estimate was also statistically imprecise (HR, 0.96; 95% CI, 0.46–1.99; *p* = 0.903; Figure 3).

Due to substantial differences in patient characteristics (see the “Before PS weighting” section of Supplementary Table S1), study settings, prior treatments, and DB exposure, the results of the unadjusted (naïve) indirect comparison should be interpreted with extreme caution because of the high potential for confounding bias.

#### 3.4.2. Exploratory Conditional Post-EOT Landmark Analysis with Adjustment for DB Exposure

A total of 80 patients initiated DB treatment across both studies. Of these, 19 experienced an EFS event on or before the landmark time point, defined as 25 days after the last DB administration. This included nine patients (25%) in the no-RA cohort and 10 patients (22.7%) in the RA cohort. The remaining 61 patients were included in the post-landmark survival analysis. This exploratory analysis included only patients who remained alive and EFS-event-free through the post-EOT landmark and therefore estimated survival within a selected conditional risk set. All patients, including those who experienced an event before the landmark, were included in the estimation of IPSWs.

After weighting, most measured characteristics appeared broadly similar; however, residual imbalance persisted, particularly for five-cycle MSK m.N7 chemotherapy (Table 3). This indicates that the post-landmark weighted comparison did not fully remove measured confounding.

In the exploratory conditional post-EOT analysis adjusted for DB exposure, the estimates remained imprecise for EFS (RA versus no RA: HR, 1.24; 95% CI, 0.37–4.12; *p* = 0.725) and OS (HR, 0.44; 95% CI, 0.07–2.71; *p* = 0.374; Figure 4). These estimates target patients who reached the landmark without an EFS event and should not be interpreted as an intention-to-treat comparison or as confirmation of the base-case analysis.

#### 3.4.3. Other Sensitivity Analyses

The evaluated sensitivity analyses produced imprecise estimates (Supplementary Table S6). No sensitivity-analysis scenario showed a clear or precisely estimated difference in EFS or OS between the RA and no-RA cohorts; all estimates should be interpreted in light of the limited effective sample size and potential residual confounding.

## 4. Discussion

In this exploratory population-adjusted comparison, no clear difference in EFS or OS was detected between the two study-defined, IL-2-free DB treatment cohorts. The primary and conditional landmark estimates were imprecise, and their confidence intervals were compatible with clinically important benefit or harm. Accordingly, the analysis did not provide evidence of more favorable survival outcomes in the RA cohort than in the no-RA cohort, but neither did it establish equivalence or demonstrate the absence of benefit from RA.

The primary overlap-weighted analysis yielded HRs of 0.92 (95% CI, 0.31–2.73) for EFS and 1.95 (95% CI, 0.46–8.20) for OS. The exploratory conditional post-EOT analysis yielded HRs of 1.24 (95% CI, 0.37–4.12) for EFS and 0.44 (95% CI, 0.07–2.71) for OS. The point estimates differed in direction across outcomes and analyses, while all intervals were wide. Nonsignificant *p* values should therefore not be interpreted as demonstrating equivalence or the absence of a clinically relevant difference between the cohorts.

The question remains biologically credible. RA can promote neuroblastoma differentiation and growth arrest through retinoid-receptor pathways, whereas DB recruits immune effector mechanisms against GD2-expressing cells [7]. Preclinical work with all-trans retinoic acid and Hu14.18K322A further suggests that retinoid-induced differentiation can preserve GD2 expression in most tested models and enhance antibody-dependent cellular cytotoxicity [10]. Our clinical analysis did not measure tumor GD2 density, RA pharmacokinetics or metabolites, MYCN modulation, RAR-pathway activity, NK-cell phenotype, or antibody-dependent cytotoxicity. It therefore cannot test or refute this proposed mechanism. Moreover, the preclinical evidence does not directly establish synergy between isotretinoin and DB.

Our findings should be interpreted in the context of the historical role of retinoid therapy in neuroblastoma. In the randomized Children’s Cancer Group 3891 trial, conducted in children with high-risk neuroblastoma who had achieved at least a partial response after induction therapy and completed consolidation without disease progression, post-consolidation RA improved 3-year EFS compared with no further therapy (46% vs. 29%; *p* = 0.027) after a median follow-up of 36 months [8]. However, with longer follow-up (median 7.7 years), the magnitude of benefit decreased and was no longer statistically significant (5-year EFS: 42% vs. 31%; *p* = 0.122), while no statistically significant difference in OS was observed (5-year OS: 50% vs. 39%; *p* = 0.195) [8,9]. This study supported the incorporation of RA into earlier multimodal treatment paradigms for newly diagnosed high-risk neuroblastoma but predated the current era of anti-GD2 maintenance therapy. Consequently, it did not address whether RA provides additional benefit when administered alongside a GD2-directed antibody backbone such as dinutuximab beta. The present study sought to address this clinically relevant question.

The present findings are also consistent with the broader evolution of anti-GD2 immunotherapy. In the randomized HR-NBL1/SIOPEN trial, the addition of subcutaneous IL-2 to DB and RA increased toxicity without improving EFS [6]. This finding shifted clinical practice toward IL-2-free treatment regimens whenever feasible, making the present comparison particularly relevant to contemporary clinical practice.

The study-dependent missingness of baseline performance status represents an important limitation. Performance status was unavailable for 15/44 patients in the RA cohort but was available for all patients in the no-RA cohort. Because missingness was completely aligned with the source study, neither complete-case analysis with this variable nor conventional imputation would provide a reliable solution. Complete-case analysis would selectively remove a substantial proportion of the RA cohort, whereas deterministic assignment or model-based imputation would require strong and unverifiable assumptions about the unobserved distribution. We therefore did not adjust for or impute the missing performance-status values. Accordingly, residual confounding related to baseline functional status cannot be excluded.

An important strength of the present study is its methodological design. The question addressed is particularly susceptible to guarantee-time and immortal-time bias because exposure to both DB and RA accumulates over time, whereas early disease progression, relapse, or death shortens treatment duration [12,14,15]. The analysis incorporated weighting and landmark approaches intended to mitigate these biases, although these methods cannot eliminate residual confounding or selection bias. In the base-case analysis, all patients were included and PS weighting was applied without adjustment for DB exposure, because DB exposure accrued after treatment initiation and was not a baseline covariate. Given the previously demonstrated association between the extent of DB exposure and both EFS and OS [19], the robustness of the base-case analysis was further evaluated using exploratory post-EOT landmark analyses in which DB exposure was defined before the landmark. These analyses should be interpreted with caution because the PS-weighted and post-landmark approaches target partially overlapping, but not identical, patient populations. The former includes all eligible patients from treatment initiation and more closely approximates an intention-to-treat comparison, whereas the latter is restricted to patients who survived and remained event-free until the landmark time point. The absence of statistically distinguishable associations across approaches reflects broadly similar statistical uncertainty; it should not be interpreted as directional consistency because the point estimates differed and the analyses targeted different populations. Neither approach fully eliminates residual confounding, exposure-related bias, or selection bias.

Use of prospectively collected individual patient data allow alignment of eligibility definitions, index dates, and measured baseline covariates. Overlap weighting also made the target population explicit and reduced some measured imbalance.

Several limitations should be emphasized. First, this was an unanchored cross-trial comparison. RA use was perfectly aligned with the source study, so protocol, eligibility, assessment schedule, calendar-period, supportive-care, and subsequent-treatment differences are inseparable from the RA contrast. Overlap weighting reduced some measured patient-level imbalance but cannot control unmeasured factors or remove study-level confounding. The estimates therefore compare two trial-defined treatment strategies, and any between-cohort differences cannot be attributed independently to RA.

Second, the nominal sample was small and weighting further reduced the effective amount of information. The cohort-specific ESSs were 10.15 for no RA and 15.30 for RA, compared with 36 and 44 patients, respectively. Patients whose baseline profiles were poorly represented in the other study received little weight, so a smaller effective amount of data informed the estimates. The sum of the two cohort-specific ESSs (25.45) is descriptive only and is not a pooled sample size or a complete determinant of variance. Cohort-specific base-case EFS-event counts, death counts, and potential follow-up estimates were not available in the validated analytical output and are therefore not reported; this limits descriptive interpretation of the survival curves. The resulting wide confidence intervals cannot exclude clinically important benefit or harm, and lack of statistical significance is not evidence of equivalence or absence of a between-cohort difference.

Third, post-baseline anticancer therapy also differed between cohorts (Supplementary Table S1). Cytotoxic chemotherapy was recorded as the first subsequent therapy in 9/44 patients in the RA cohort and in 0/36 patients in the no-RA cohort, whereas retinoid/transplant-related consolidation was recorded in 1/44 and 4/36 patients, respectively. These differences may reflect protocol, disease course, and clinical decision-making and could materially influence OS after the initial DB strategy. Because subsequent therapy occurs after baseline and may be a mediator or consequence of progression, it was not included as a baseline confounder; nevertheless, the imbalance further limits attribution of OS differences to RA use. Outcome ascertainment was also not fully identical between the source studies. APN311-304 originally reported PFS, and information on secondary neoplasms was not available in the extracted dataset, whereas APN311-202 V3 used EFS. The harmonized endpoint therefore relied on the event components available in both studies. Although secondary neoplasms are expected to be uncommon in this clinical setting, this difference represents an additional limitation of cross-study endpoint harmonization.

The post-EOT landmark analysis was exploratory and conditional on patients remaining alive and EFS-event-free through the landmark. It therefore describes a selected post-treatment risk set and should not be interpreted as an intention-to-treat comparison or as confirmation of the base-case analysis.

Independent reviews emphasize that neuroblastoma treatment is risk-adapted and multimodal, with therapeutic strategies shaped by disease stage, tumor biology, prior therapy, consolidation, and maintenance [3,27]. This broader context reinforces that the contribution of a single maintenance component cannot be inferred reliably when that component is perfectly aligned with the source study and other elements of the treatment pathway differ between cohorts.

These limitations are consistent with methodological evidence showing that population-adjustment methods can reduce measured cross-study imbalance only under strong assumptions and that uncertainty may be understated when covariate overlap and effective sample size are limited [28]. The study-dependent missingness of performance status is particularly important because treating missingness as a category does not, by itself, recover the unobserved covariate distribution or remove bias [29]. Accordingly, interpretation should focus on the effect estimates and their confidence intervals rather than on whether individual *p* values cross a conventional threshold [30].

Safety was not compared between the treatment cohorts because adverse event collection, grading practices, and monitoring intensity differed between the study protocols. This limitation is particularly relevant for RA, for which the detection and reporting of mucocutaneous toxicities and laboratory abnormalities may be influenced by protocol-specific monitoring procedures.

Clinical decisions about RA should not be based on this indirect comparison alone. They should integrate evidence from other disease settings, the treatment protocol and its intended maintenance strategy, disease status and prior therapy, expected RA-related toxicity and treatment burden, and individual patient circumstances. Accordingly, the present findings neither support routine omission of RA nor establish that RA should be continued in every patient.

The present results should be used to refine, rather than settle, the clinical question. A prospective component-randomized design would provide the clearest estimate of the incremental contribution of RA. If randomization is not feasible, a prospectively specified multicenter registry analysis should use a common protocol era, harmonized assessments, detailed salvage-treatment data, and embedded biomarkers of retinoid exposure, GD2 expression, and immune-effector function. Safety and patient burden should be measured with identical schedules because any small survival effect must be interpreted alongside RA-associated toxicity and treatment burden.

## 5. Conclusions

In this exploratory unanchored comparison of two prospective cohorts comprising 80 patients with relapsed or refractory neuroblastoma, no clear difference in EFS or OS was detected between the overlap-weighted, study-defined IL-2-free DB treatment strategies with and without RA. The analysis did not provide clear evidence of more favorable survival outcomes in the RA cohort than in the no-RA cohort. However, the estimates were highly imprecise and compatible with clinically important benefit or harm. Because RA use was inseparable from the source study, covariate overlap and cohort-specific effective sample sizes were limited, performance-status missingness differed between studies, and residual patient-level and protocol-level confounding could not be excluded. The findings should therefore not be interpreted as evidence of equivalence or as evidence that RA provides no benefit. They are hypothesis-generating and should not alone guide continuation or omission of RA.

## Figures and Tables

**Figure 1:**
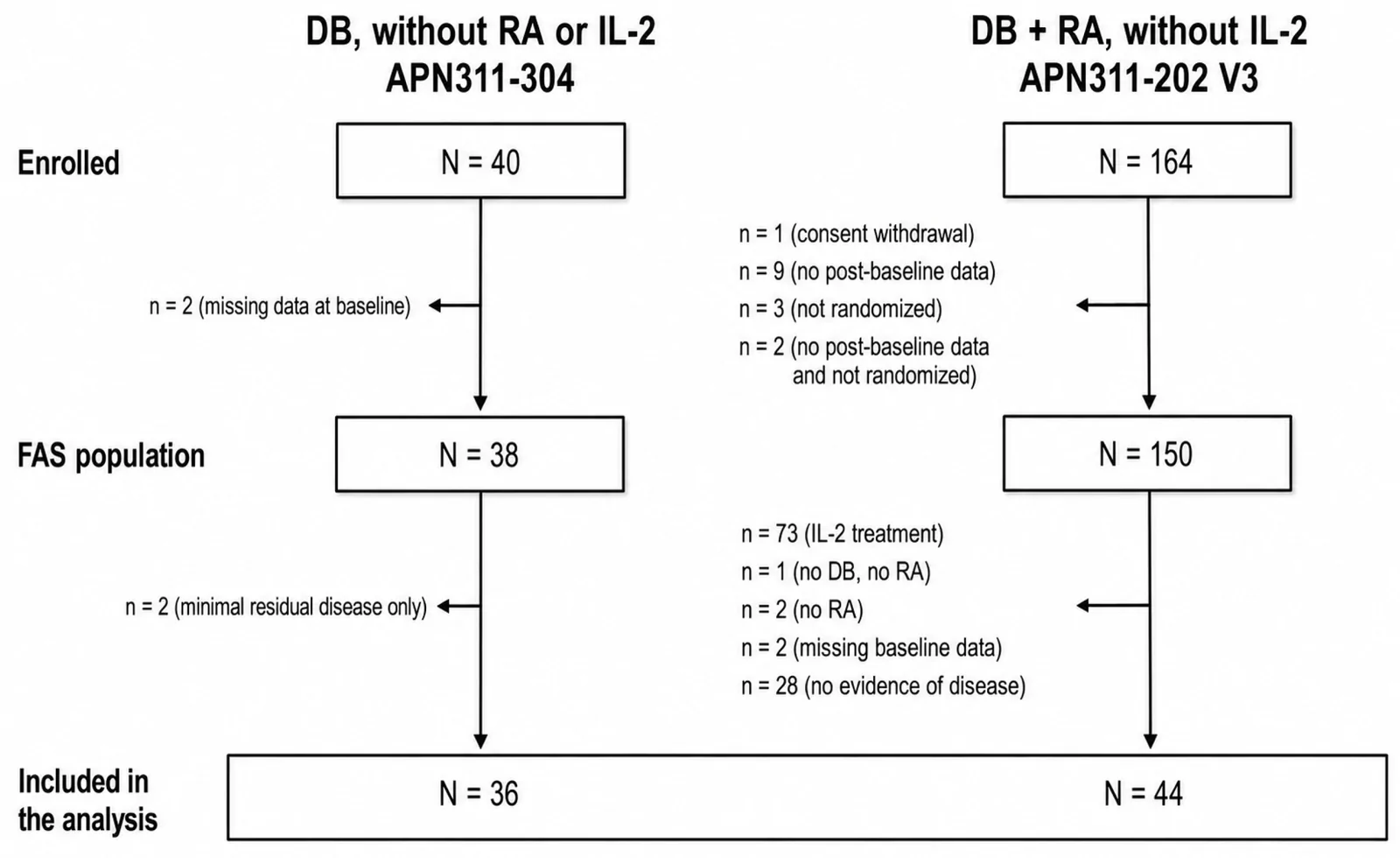
Patient selection. DB, dinutuximab beta; FAS, full analysis set; IL-2, interleukin-2; RA, 13-cis-retinoic acid.

**Figure 2:**
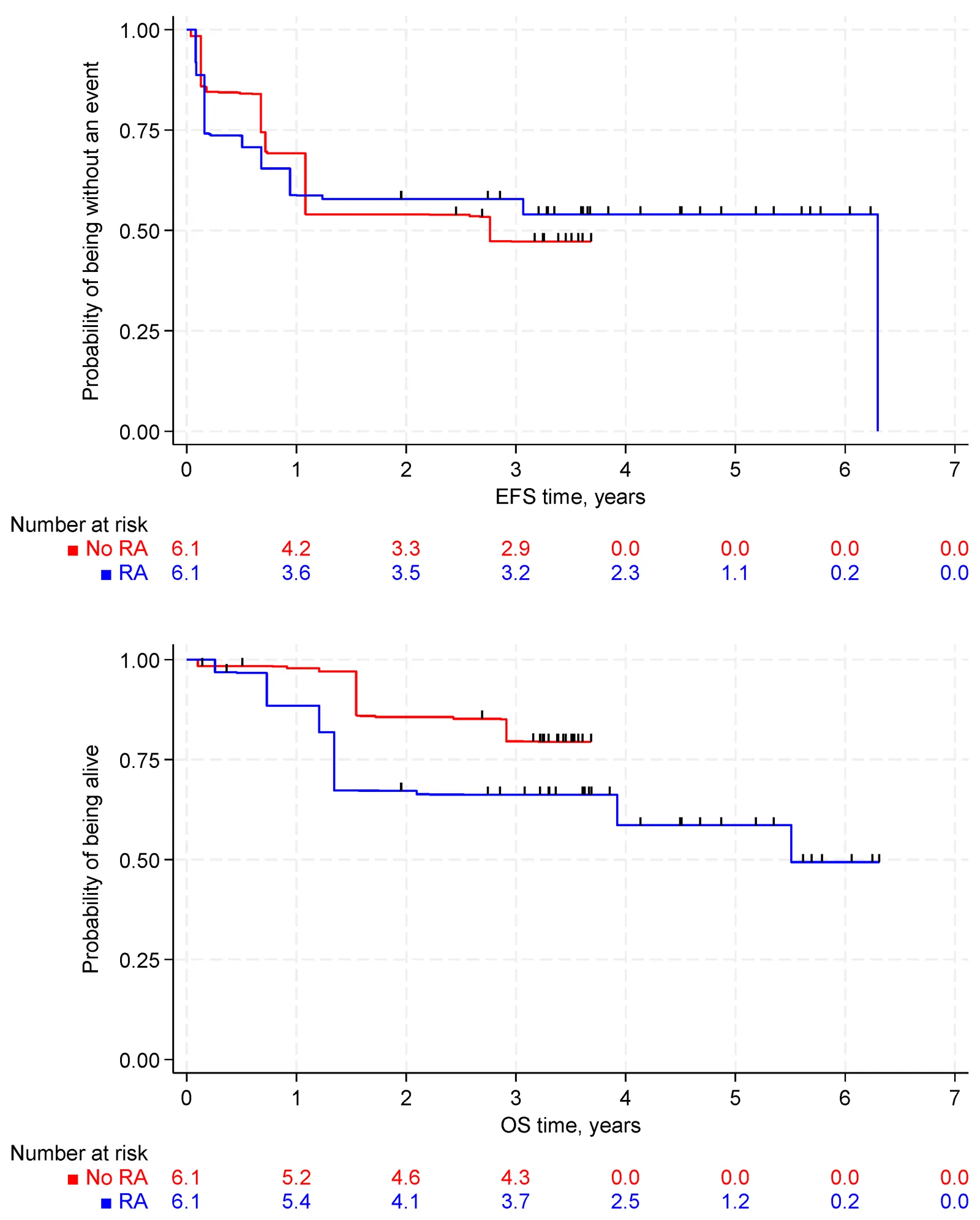
EFS (**upper panel**) and OS (**lower panel**) from the first DB administration after PS weighting. Numbers at risk are sums of overlap weights and should not be interpreted as patient counts or effective sample sizes.

**Figure 3:**
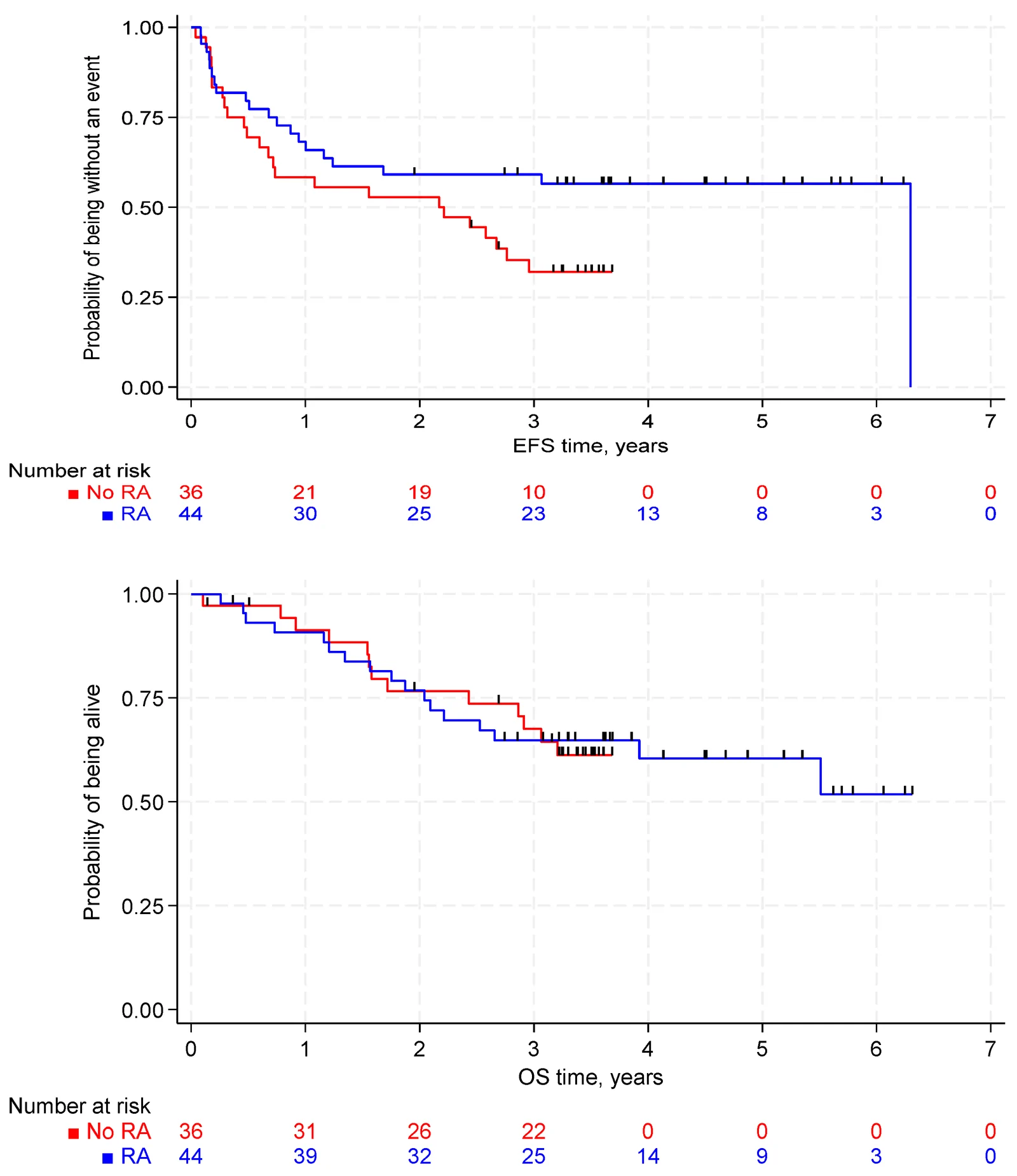
EFS (**upper panel**) and OS (**lower panel**) in the unadjusted (naïve) comparison.

**Figure 4:**
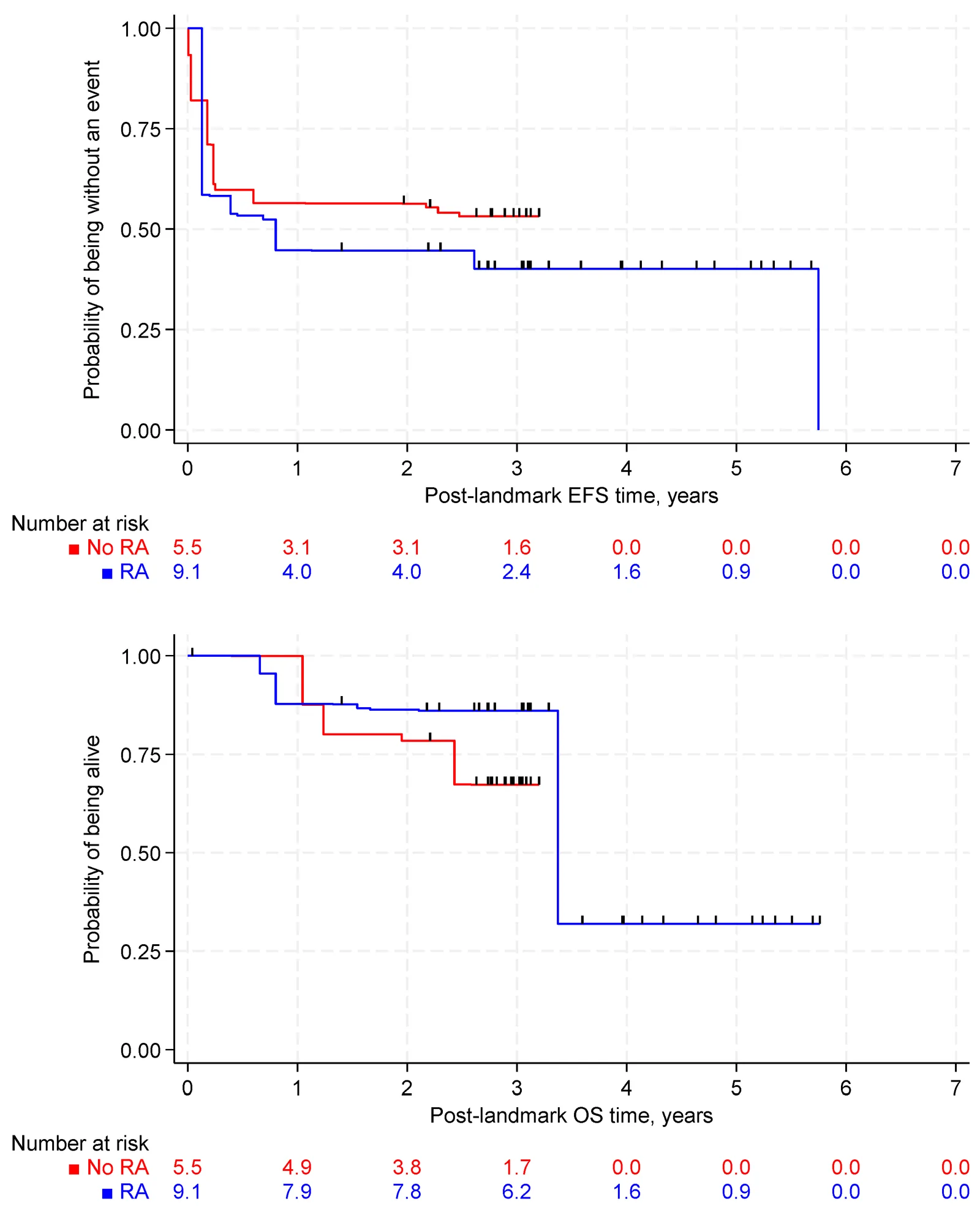
EFS (**upper panel**) and OS (**lower panel**) in the post-landmark analysis with adjustment for DB exposure. Numbers at risk are sums of the final analysis weights and should not be interpreted as patient counts or effective sample sizes.

**Table 1:** Main prospective and protocol-defined DB cohorts considered for the assessment of treatment strategies with or without RA.

Study, Cohort	Comment
Included studies	
APN311-304 [2]	Relapsed/refractory indication DB dosing schedule matched the target regimen Prospective design
APN311-202, V3 (stage II) [20]	
Excluded studies	
APN311-303 (compassionate use) [20]	Retrospective design IL-2 coadministration DB: Up to 6 35-day cycles Compassionate-use setting with potential nonrepresentativeness and incompletely specified treatment or patient-selection processes
APN311-202, V1 + V2 (stage I) [20]	IL-2 coadministration in all patients
APN311-302 R2/R4 [6]	Frontline maintenance immunotherapy No IPD available from APN311-302 R4
Wieczorek et al. 2023 [21]	Retrospective chart-review design. Mixed first-line and relapsed/refractory population.
APN311-201 (Flaadt et al. 2023) [22]	Dosing differed from the target regimen (28-day cycle) Mandatory haploidentical SCT defined a different treatment pathway and population
Mueller et al. 2018 [23]	No information on disease status before DB treatment was reported.
APN311-101 (Ladenstein et al. 2013) [20]	Dosing differed from the target regimen (28-day cycle; maximum of three cycles; only 10 patients received the recommended dose) Retrospective design

DB—dinutuximab beta; IL-2—interleukin-2; IPD—individual patient data; SCT—stem cell transplantation.

**Table 2:** Harmonized inclusion and exclusion criteria for the indirect comparison.

	Inclusion Criteria	Exclusion Criteria
Base-case analysis	(1) Age ≥ 12 months and ≤21 years at study entry (2) Diagnosis: neuroblastoma per INSS (International Neuroblastoma Staging System). (3) Primary refractory or relapsed neuroblastoma consistent with protocol eligibility; patients with minimal residual disease (2 patients) only in APN311-304 were excluded for consistency. (4) Measurable/evaluable disease at entry (5) Performance status ≥ 70% (Lansky/Karnofsky) (6) Life expectancy ≥ 12 weeks (7) Off standard/experimental anti-cancer treatment ≥ 2 weeks and recovered from short-term major toxicities (8) No immediate requirement for palliative chemotherapy/radiotherapy/surgery (9) ≥2 weeks after any surgery and recovered (10) HIV negative and no active/chronic replicative hepatitis B infection (11) Pregnancy prevention requirements per protocol (12) Prior CNS metastases allowed only if previously treated, clinically stable ≥ 4 weeks, and off steroids for CNS disease ≥ 4 weeks before and during study (13) Seizure disorder allowed if controlled (and, where recorded, no seizures in the last 6 weeks) (14) Shortening fraction ≥ 30% (15) Pulmonary function: FEV1 and FVC > 60% predicted; if unable to perform PFTs: no dyspnoea at rest and SpO2 ≥ 94% room air (16) Adequate marrow/liver/renal function (17) DB treatment (at least one dose) without IL-2 coadministration	(1) No disease measurable by MRI or CT and no disease evaluable by MIBG or technetium scan or BM histology (2) Progressive disease at inclusion (3) Requires or likely requires corticosteroids or other immunosuppressive drugs (4) Platelet transfusion dependent (5) Significant intercurrent illness
Additional exploratory restrictions evaluated in sensitivity analyses	-	(1) Prior anti-GD2 antibody exposure (4 patients from Study 304) (2) Post-baseline receipt of selected subsequent therapies, evaluated only in a previously performed exploratory exclusion analysis and not treated as a baseline eligibility criterion or confounder

CNS, central nervous system; CT, computed tomography; DB, dinutuximab beta; FEV_1_, forced expiratory volume in 1 s; FVC, forced vital capacity; HIV, human immunodeficiency virus; IL-2, interleukin-2; INSS, International Neuroblastoma Staging System; MIBG, metaiodobenzylguanidine; MRI, magnetic resonance imaging; SpO_2_, peripheral oxygen saturation. Protocol eligibility required a performance status score of at least 70; however, the numerical baseline score was unavailable in the analytical dataset for 15/44 patients in the RA cohort.

**Table 3:** Characteristics of the post-landmark study population by cohort.

Characteristic			Weighted Post-Landmark Population ^A^		
			No RA (N = 27, Σw = 5.5)	RA (N = 34, Σw = 9.1)	*p* Value
Age at DB initiation		Median (IQR)	7.6 (4.7 to 8.0)	5.4 (5.0 to 7.0)	0.763
		Mean (SE)	7.0 (0.8)	6.6 (1.2)	
Age at diagnosis		Median (IQR)	5.1 (2.8 to 6.6)	3.0 (3.0 to 4.5)	0.400
		Mean (SE)	5.1 (0.7)	4.2 (0.9)	
Months since diagnosis		Median (IQR)	17.0 (13.0 to 30.0)	29.4 (20.3 to 29.4)	0.965
		Mean (SE)	29.0 (6.2)	29.3 (4.4)	
Year of the diagnosis		Median (IQR)	2015 (2014 to 2015)	2013.0 (2013 to 2014)	By design (0.190)
		Mean (SE)	2014.0 (0.6)	2013.0 (0.4)	
Sex	Male	n (%)	3.7 (66.3%)	4.4 (80.3%)	0.431
	Female		1.9 (33.8%)	1.1 (19.7%)	
Ethnicity	Asian	n (%)	0.0 (0.0%)	0.3 (3.8%)	0.679
	Black		0.0 (0.0%)	0.0 (0.5%)	
	Italian/Philippines		0.0 (0.0%)	0.0 (0.1%)	
	not available		0.0 (0.0%)	0.4 (3.9%)	
	White or Caucasian		5.5 (100.0%)	8.1 (89.6%)	
	Cuban		0.0 (0.0%)	0.2 (2.0%)	
Baseline performance score B	70	n (%)	0.0 (0.0%)	0.0 (0.0%)	0.676
	80		0.1 (1.4%)	0.0 (0.0%)	
	90		1.8 (32.5%)	2.2 (24.1%)	
	100		3.6 (66.1%)	6.9 (75.9%)	
Baseline MIBG scan	soft tissue lesions	n (%)	2.2 (39.0%)	3.5 (62.7%)	0.320
	distant lymph nodes	n (%)	0.0 (0.0%)	0.2 (4.4%)	0.433
	liver lesions	n (%)	0.4 (6.6%)	0.3 (5.9%)	0.925
	skeletal lesions	n (%)	3.6 (66.1%)	4.2 (75.4%)	0.639
INSS at diagnosis	INSS = 4	n (%)	4.2 (75.7%)	4.5 (82.3%)	0.706
	other		1.3 (24.3%)	1.0 (17.7%)	
Disease status	Relapsed	n (%)	2.9 (52.7%)	3.7 (67.8%)	0.495
	Refractory		2.6 (47.3%)	1.8 (32.3%)	
*MYCN*	Other *MYCN*	n (%)	5.1 (92.0%)	5.1 (92.6%)	0.944
	Amplified		0.4 (8.0%)	0.4 (7.4%)	
Frontline treatment	Chemotherapy	n (%)	4.8 (87.0%)	5.3 (95.9%)	0.315
	High-dose therapy	n (%)	3.6 (66.0%)	4.4 (79.1%)	0.471
	Local therapy	n (%)	5.0 (91.3%)	4.7 (84.3%)	0.560
	Maintenance therapy	n (%)	1.5 (27.7%)	2.9 (52.6%)	0.307
	Radiotherapy	n (%)	2.7 (48.5%)	3.6 (64.7%)	0.487
	Surgery	n (%)	4.1 (74.7%)	4.6 (84.1%)	0.553
	Anti-GD2 maintenance	n (%)	0.1 (1.5%)	0.0 (0.0%)	0.214
	High-dose therapy: MIBG	n (%)	1.3 (23.0%)	0.9 (16.2%)	0.674
	High-dose therapy: BuMEL	n (%)	2.8 (50.9%)	3.7 (67.6%)	0.460
	High-dose therapy: CEM	n (%)	0.3 (5.2%)	0.2 (3.8%)	0.814
	Chemotherapy: rapid COJEC	n (%)	2.2 (39.7%)	3.4 (62.0%)	0.359
	Chemotherapy: MSK m.N7 (5 cycles)	n (%)	0.1 (1.5%)	1.6 (28.6%)	0.001
Cycles with DB dose of at least 50 mg/m^2^	1	n (%)	0.6 (11.3%)	0.0 (0.0%)	0.380
	2		0.0 (0.0%)	0.6 (6.5%)	
	3		0.0 (0.0%)	1.1 (11.6%)	
	4		0.0 (0.0%)	0.0 (0.4%)	
	5		4.9 (88.7%)	7.4 (81.5%)	
Initiated DB treatment cycles [19]	1	n (%)	0.6 (11.3%)	0.0 (0.0%)	0.417
	2		0.0 (0.0%)	0.5 (5.0%)	
	3		0.0 (0.0%)	0.7 (7.7%)	
	4		0.0 (0.0%)	0.4 (3.9%)	
	5		4.9 (88.7%)	7.5 (83.3%)	
Cumulative DB dose (mg/m^2^)		Median (IQR)	468.0 (461.5 to 481.0)	493.0 (430.2 to 493.0)	0.650
		Mean (SE)	428.3 (40.0)	449.9 (25.6)	
First subsequent anticancer therapy	Local therapy	n (%)	1.7 (30.5%)	1.6 (17.4%)	Inapplicable due to conditioning on subsequent treatment (0.091)
	Retinoid/transplant consolidation		3.8 (69.5%)	1.3 (14.3%)	
	Targeted/biologic systemic therapy		0.0 (0.0%)	0.3 (2.8%)	
	Cytotoxic chemotherapy		0.0 (0.0%)	5.9 (65.5%)	

^A^ Σw—Values are weighted estimates. The symbol w denotes the sum of weights and should not be interpreted as the effective sample size. BuMEL—busulfan and melphalan; CEM—carboplatin, etoposide, melphalan; COJEC—cisplatin, vincristine, carboplatin, etoposide, cyclophosphamide; DB—dinutuximab beta; INSS—International Neuroblastoma Staging System; IQR—interquartile range; MIBG—metaiodobenzylguanidine; MSK m.N7—Memorial Sloan Kettering modified N7 protocol; *MYCN*—*MYCN* oncogene; PS—propensity score; RA—isotretinoin (13-cis-retinoic acid); SD—standard deviation; SE—standard error. B Baseline performance status was not included in the base-case propensity-score model because missingness was study-dependent. The post-landmark values are presented descriptively among patients with observed performance status only and should not be interpreted as evidence of covariate balance. No imputation was performed.

## Data Availability

The data presented in this study are available on request from the corresponding author, subject to the original trial agreements and applicable privacy requirements. The data are not publicly available because they contain de-identified individual patient data governed by those agreements and requirements.

## Supplementary Materials

The following supporting information can be downloaded at https://www.mdpi.com/article/10.3390/biomedicines14102200/s1.

## Abbreviations

The following abbreviations are used in this manuscript:

13-cis-RA	13-cis-retinoic acid
ADA	anti-drug antibody
BM	bone marrow
BuMEL	busulfan and melphalan
CEM	carboplatin, etoposide, and melphalan
CI	confidence interval
COJEC	cisplatin, vincristine, carboplatin, etoposide, and cyclophosphamide
CT	computed tomography
DB	dinutuximab beta
EFS	event-free survival
EOT	end of treatment
FAS	full analysis set
HR	hazard ratio
IL-2	interleukin-2
INSS	International Neuroblastoma Staging System
IPD	individual patient data
IPSW	inverse probability of selection weights
IQR	interquartile range
LDH	lactate dehydrogenase
MIBG	metaiodobenzylguanidine
MRI	magnetic resonance imaging
OS	overall survival
PS	propensity score
RA	13-cis-retinoic acid (isotretinoin)
R/R	relapsed or refractory
SA	sensitivity analysis/analyses
SD	standard deviation
