# Supplementary material for: Survival Outcomes with IL-2-Free Dinutuximab Beta Treatment Strategies With or Without 13-cis-Retinoic Acid in Relapsed or Refractory Neuroblastoma: An Exploratory Unanchored, Population-Adjusted Comparison of Two Prospective Cohorts
Source: Biomedicines. 2026 Sep 29;14(10):2200. doi: 10.3390/biomedicines14102200 (PMC13701175; doi:10.3390/biomedicines14102200)
Supplement: Supplementary file 1 [file biomedicines-14-02200-s001.zip › biomedicines-4515225-supplementary.pdf]

Supplementary materials

Supplementary Table S1. Characteristics of the study population by cohort (overall study population).

Characteristic			Before PS weighting			After PS weighting [A]		
			**No RA (N=36)**	**RA (N=44)**	**P value**	**No RA (Σw=6.1)**	**RA (Σw=6.1)**	**P value**
**Age at DB initiation**		Median (IQR)	6.8 (4.7 to 8.1)	5.3 (3.8 to 7.8)	0.226	7.1 (4.1 to 8.0)	5.4 (4.7 to 7.9)	0.999
		Mean (SD/SE)	7.0 (3.4)	6.5 (4.0)		6.5 (0.6)	6.5 (0.9)	
**Age at diagnosis**		Median (IQR)	4.0 (2.9 to 5.5)	3.2 (2.3 to 5.3)	0.128	3.6 (2.6 to 6.6)	3.2 (2.5 to 5.4)	0.654
		Mean (SD/SE)	4.5 (2.3)	4.2 (3.3)		4.6 (0.8)	4.2 (0.6)	
**Months since diagnosis**		Median (IQR)	25.0 (16.0 to 43.5)	18.6 (13.8 to 27.6)	0.107	22.0 (13.0 to 30.0)	22.6 (17.6 to 30.0)	0.998
		Mean (SD/SE)	35.8 (34.3)	27.9 (25.4)		28.0 (5.8)	28.0 (4.3)	
**Year of the diagnosis**		Median (IQR)	2014 (2012 to 2015)	2014 (2013 to 2014)	By design (0.252)	2014 (2014 to 2015)	2013 (2012 to 2014)	By design (0.214)
		Mean (SD/SE)	2013.3 (3.1)	2013.0 (2.3)		2013.9 (0.7)	2012.8 (0.5)	
**Sex**	Male	n (%)	25 (69.4%)	25 (56.8%)	0.246	3.8 (62.2%)	3.8 (62.1%)	0.997
	Female		11 (30.6%)	19 (43.2%)		2.3 (37.9%)	2.3 (37.9%)	
**Ethnicity**	Asian	n (%)	0 (0%)	2 (4.5%)	0.401	0.0 (0.0%)	0.3 (4.9%)	0.392
	Black		0 (0%)	2 (4.5%)		0.0 (0.0%)	0.0 (0.2%)	
	Italian/ Philippines		0 (0%)	1 (2.3%)		0.0 (0.0%)	0.0 (0.0%)	
	not available		0 (0%)	2 (4.5%)		0.0 (0.0%)	0.2 (3.5%)	
	White or Caucasian		36 (100%)	36 (81.8%)		6.1 (100.0%)	5.4 (88.2%)	
	Cuban		0 (0%)	1 (2.3%)		0.0 (0.0%)	0.2 (3.2%)	
**Baseline performance score**	70	n (%)	1 (2.8%)	0 (0%)	**0.001** (0.198 without 'missing' level)	0.1 (1.6%)	0.0 (0.0%)	**0.037**
	80		2 (5.6%)	0 (0%)		0.4 (6.7%)	0.0 (0.0%)	
	90		15 (41.7%)	8 (18.2%)		2.9 (47.7%)	0.5 (8.5%)	

Characteristic			Before PS weighting			After PS weighting [A]		
			No RA (N=36)	RA (N=44)	P value	No RA (Σw=6.1)	RA (Σw=6.1)	P value
	100		18 (50%)	21 (47.7%)		2.7 (44.1%)	5.6 (91.5%)	
	missing		0 (0%)	15 (34.1%)		-	-	
**Baseline MIBG scan**	soft tissue lesions	n (%)	12 (33.3%)	22 (50%)	0.134	2.0 (32.1%)	2.0 (32.1%)	0.997
	distant lymph nodes	n (%)	0 (0%)	5 (11.4%)	**0.037**	0.0 (0.0%)	0.4 (5.8%)	0.229
	liver lesions	n (%)	2 (5.6%)	5 (11.4%)	0.360	0.0 (0.7%)	0.2 (3.3%)	0.087
	skeletal lesions	n (%)	27 (75%)	28 (63.6%)	0.275	4.5 (73.5%)	4.5 (73.5%)	0.997
**INSS at diagnosis**	INSS=4	n (%)	33 (91.7%)	35 (79.5%)	0.131	5.0 (82.0%)	5.0 (82.0%)	0.997
	other		3 (8.3%)	9 (20.5%)		1.1 (18.0%)	1.1 (18.1%)	
**Disease status**	Relapsed	n (%)	18 (50%)	14 (31.8%)	0.099	3.6 (59.1%)	3.6 (59.1%)	0.999
	Refractory		18 (50%)	30 (68.2%)		2.5 (41.0%)	2.5 (41.0%)	
MYCN	Other *MYCN*	n (%)	31 (86.1%)	35 (79.5%)	0.442	5.6 (91.7%)	5.6 (91.6%)	0.998
	Amplified		5 (13.9%)	9 (20.5%)		0.5 (8.3%)	0.5 (8.4%)	
**Frontline treatment**	Chemotherapy	n (%)	34 (94.4%)	42 (95.5%)	0.837	5.8 (94.5%)	5.8 (95.1%)	0.933
	High-dose therapy	n (%)	27 (75%)	27 (61.4%)	0.195	4.8 (77.8%)	4.9 (79.3%)	0.920
	Local therapy	n (%)	34 (94.4%)	30 (68.2%)	**0.003**	5.5 (89.2%)	5.2 (84.2%)	0.712
	Maintenance therapy	n (%)	25 (69.4%)	8 (18.2%)	**<0.001**	2.4 (39.3%)	2.4 (39.2%)	0.998
	Radiotherapy	n (%)	13 (36.1%)	23 (52.3%)	0.148	3.0 (48.5%)	3.0 (48.5%)	0.999
	Surgery	n (%)	32 (88.9%)	25 (56.8%)	**0.002**	5.2 (84.2%)	5.2 (84.1%)	0.995
	Anti-GD2 maintenance	n (%)	3 (8.3%)	0 (0%)	0.051	0.0 (0.8%)	0.0 (0.0%)	0.240
	High-dose therapy: MIBG	n (%)	13 (36.1%)	8 (18.2%)	0.070	1.0 (16.7%)	1.0 (16.6%)	0.998
	High-dose therapy: BuMEL	n (%)	12 (33.3%)	24 (54.5%)	0.058	4.4 (71.6%)	4.4 (71.6%)	0.999
	High-dose therapy: CEM	n (%)	12 (33.3%)	2 (4.5%)	**0.001**	0.2 (3.1%)	0.5 (7.7%)	0.325
	Chemotherapy: rapid COJEC	n (%)	5 (13.9%)	29 (65.9%)	**<0.001**	3.3 (54.5%)	3.3 (54.5%)	0.998
	Chemotherapy: MSK m.N7 (5 cycles)	n (%)	2 (5.6%)	10 (22.7%)	**0.032**	0.0 (0.8%)	2.1 (33.6%)	**<0.001**
	1	n (%)	2 (5.6%)	2 (4.5%)	0.648	0.9 (14.1%)	0.7 (11.3%)	0.102
	2		4 (11.1%)	7 (15.9%)		0.1 (1.4%)	1.4 (22.8%)	

Characteristic			Before PS weighting			After PS weighting [A]		
			No RA (N=36)	RA (N=44)	P value	No RA (Σw=6.1)	RA (Σw=6.1)	P value
**Cycles with DB dose of at least 50 mg/m^2**	3		1 (2.8%)	3 (6.8%)		0.0 (0.1%)	0.2 (3.8%)	
	4		3 (8.3%)	1 (2.3%)		0.0 (0.1%)	0.0 (0.1%)	
	5		26 (72.2%)	31 (70.5%)		5.2 (84.3%)	3.8 (62.1%)	
**Initiated DB treatment cycles [19]**	1	n (%)	2 (5.6%)	2 (4.5%)	0.928	0.9 (14.1%)	0.7 (11.3%)	0.164
	2		4 (11.1%)	6 (13.6%)		0.1 (1.4%)	1.1 (18.3%)	
	3		1 (2.8%)	2 (4.5%)		0.0 (0.1%)	0.0 (0.3%)	
	4		2 (5.6%)	1 (2.3%)		0.0 (0.1%)	0.2 (3.5%)	
	5		27 (75%)	33 (75%)		5.2 (84.3%)	4.1 (66.6%)	
**Cumulative DB dose (mg/m^2)**		Median (IQR)	466.1 (396.7 to 476.5)	474.6 (328.9 to 497.4)	0.178	466.3 (406.7 to 479.5)	430.2 (199.4 to 495.3)	0.505
		Mean (SD/SE)	405.9 (121.6)	404.5 (127.2)		407.5 (43.5)	367.8 (40.3)	
**First subsequent anticancer therapy**	Local therapy	n (%)	1 (2.8%)	4 (9.1%)	Descriptive only; not interpreted because subsequent therapy is a post-baseline variable (available legacy P value: 0.005)	1.7 (28.4%)	0.8 (12.6%)	Inapplicable due to conditioning on subsequent treatment (**0.006**)
	Retinoid / transplant consolidation		4 (11.1%)	1 (2.3%)		4.4 (71.6%)	0.2 (3.8%)	
	Targeted / biologic systemic therapy		0 (0%)	3 (6.8%)		0.0 (0.0%)	0.0 (0.3%)	
	Cytotoxic chemotherapy		0 (0%)	9 (20.5%)		0.0 (0.0%)	5.1 (83.4%)	

[A] Σw – values after weighting are overlap-weighted estimates. The symbol w denotes the sum of weights and should not be interpreted as the effective sample size. The symbol w denotes the sum of weights and should not be interpreted as the effective sample size. BuMEL – busulfan and melphalan; CEM – carboplatin, etoposide, melphalan; COJEC – cisplatin, vincristine, carboplatin, etoposide, cyclophosphamide; DB – dinutuximab beta; INSS – International Neuroblastoma Staging System; IQR – interquartile range; MIBG – metaiodobenzylguanidine; MSK m.N7 – Memorial Sloan Kettering modified N7 protocol; *MYCN* – *MYCN* oncogene; PS – propensity score; RA – isotretinoin (13-cis-retinoic acid); SD – standard deviation; SE – standard error. Baseline performance status was excluded from the propensity-score model because missingness was study-dependent. Post-weighting values are presented descriptively among patients with observed performance status and should not be interpreted as evidence of balance. No imputation was performed

Supplementary Table S2. A comparison of the inclusion and exclusion criteria, study design, and planned treatment regimens between Study APN311-304 and Study APN311-202 V3

	APN311-202 V3 [20]	Study APN311-304 [2]	Comment
**Arm of interest**	Experimental arm (DB via LTI; RA, no IL-2)	All patients (DB via LTI; no RA, no IL-2)	DB plus RA without IL-2 versus DB without RA or IL-2
**Design / setting**	Prospective, open-label, randomised, multi-centre phase I/II; V3 amendment evaluates role of IL-2 for EFS	Open-label, uncontrolled, two-centre phase II	-
**Age**	>1 year and ≤21 years at entry	≥12 months and ≤21 years at entry	-
**Diagnosis**	Neuroblastoma per INSS.	Neuroblastoma per INSS.	-
**Required prior therapy / line of therapy anchor**	At least one prior high-dose treatment with stem-cell rescue after conventional therapy	No explicit requirement for "≥1 high-dose + stem-cell rescue" in inclusion criteria; eligibility anchored on current disease status + "tumour burden controlled by conventional therapy" (exception: early minimal BM relapse)	Study 304:<br>9 patients without "1st line high-dose therapy"<br>2 patients with 'minimal residual disease only'
**Disease status required at entry**	Patients had to meet the criteria for a defined high-risk context (stage 4 in the current SIOPEN HR-NBL-1(1.5), with refractory or ineligible randomisation due to delays; other intensive induction, high-dose and rescue; or treatment of responding relapse after primary stage 4, without progression at entry).	Primary refractory stage 4, or relapse after primary stage 4, or disseminated relapse after primary localized neuroblastoma, with tumour burden controlled by conventional therapy (except early minimal BM relapse)	Study 304:<br>2 patients with 'minimal residual disease only'
**Measurable/evaluable disease required**	enrolment was also permitted for patients without any signs of disease.	Requires measurable and/or evaluable disease (skeletal/soft tissue/lymph node/primary site/BM) assessed by MIBG, CT, MRI and/or immunocytology	Patients without evidence of disease in the APN311-202 V3 study
**Performance status**	≥70% (Lansky/Karnofsky)	≥70% (Lansky/Karnofsky)	-
**Life expectancy**	≥12 weeks	≥12 weeks	-
**Washout / no immediate palliative need**	Off standard/experimental treatments ≥2 weeks prior to entry; no immediate need for palliative chemo/RT/surgery; includes detailed cardiopulmonary and lab requirements	Off standard/experimental treatments ≥2 weeks prior to Day 1 Cycle 1; no immediate need for palliative chemo/RT/surgery; includes detailed cardiopulmonary and lab requirements	-
**Major surgery timing**	≥4 weeks after major surgery and recovered.	≥2 weeks from tumour surgery and recovered.	-

	APN311-202 V3 [20]	Study APN311-304 [2]	Comment
**Organ function / labs**	Required (cardiac/pulmonary and other criteria implied; full list continues beyond excerpt).	Explicit cardiac (left ventricular fractional shortening ≥30%) pulmonary (FEV1/FVC >60% or SpO_2>94%), marrow, liver, renal criteria.	-
**CNS metastases**	Allowed if previously treated, clinically stable ≥4 weeks, and off steroids for CNS disease ≥4 weeks.	Allowed with similar stability/off-steroids conditions.	-
**Key exclusions: disease activity**	Progressive disease excluded	Progressive disease excluded	-
**Key exclusions: prior anti-GD2**	Excludes patients previously treated with ch14.18/SP2/0 and/or ch14.18/CHO	Excludes ADA positivity due to prior anti-GD2 (i.e., prior anti-GD2 per se is not framed as an automatic exclusion; the exclusion is antibody positivity).	Study 304:<br>4 patients with previous maintenance therapy with anti-GD2
**Key exclusion: steroids / immunosuppression**	Patients requiring (or likely requiring) corticosteroids or other immunosuppressive drugs excluded	-	-
**Key exclusions: other clinical constraints**	Platelet transfusion–dependent; significant intercurrent illness; active infections; neuropathy > Grade 2; symptomatic pleural effusions; psychiatric/cardiac rhythm issues etc.	Likely need for corticosteroids/immunosuppressants; concurrent non-trial anticancer therapies; hypersensitivity to product/mouse proteins; pregnancy/lactation/no contraception.	-
**DB (dinutuximab beta) regimen timing within cycle**	Continuous infusion starts Day 8, planned duration 10 days (i.e., Day 8–18)<br>10 mg/m²/day over 10 days (total 100 mg/m²/cycle)	Continuous infusion Day 1–11, 10 mg/m²/day over 10 days (total 100 mg/m²/cycle)	DB starts 7-8 days earlier in Study 304 than in the APN311-202 V3 study
**IL-2**	Not part of regimen of the experimental arm	Not part of regimen	By design
**Isotretinoin (13-cis-RA)**	160 mg/m²/day (or 200 mg/m²/day for >12 kg unable to swallow capsules), BID for 14 days; starts Day 19 (after DB infusion completion).	Not part of regimen	By design
**Cycle length / number of cycles**	35-day cycles; up to 5 cycles	35-day cycles; up to 5 cycles in absence of progression	-
**Bone marrow assessment**	Tumour assessments include bone marrow (BM) among the modalities used for post-baseline assessments.	Immunocytology evaluated centrally; AIPF (“automatic immuno-fluorescence plus FISH”) as part of immunocytology work-up	-
**Concomitant / prohibited therapies**	The protocol includes extensive safety management and monitoring, and the	It explicitly prohibits the use of concurrent chemotherapy, radiotherapy and experimental anticancer medications during the on-study	-

	APN311-202 V3 [20]	Study APN311-304 [2]	Comment
	exclusion criteria include the likelihood of needing steroids or immunosuppression.	period. It also restricts the use of glucocorticoids and immunosuppressants around the date of blood draw (DB) and restricts intravenous immunoglobulin (IVIG) due to the potential for interference with antibody-dependent cellular cytotoxicity	

ADA – anti-drug antibodies; BID – *bis in die*; BM – bone marrow; CT – computed tomography; DB – dinutuximab beta; EFS – event-free survival; FEV_1 – forced expiratory volume in 1 second; FVC – forced vital capacity; IL-2 – interleukin-2; LTI – long-term infusion; MIBG – metaiodobenzylguanidine; MRI – magnetic resonance imaging; RA – isotretinoin; RT – radiotherapy; SpO_2 – peripheral oxygen saturation.

Supplementary Table S3. Original survival endpoints of each trial.

Study	Original endpoint	Endpoint information available for the present analysis
**APN311-304**	PFS (long-term analysis), OS	PFS and OS were available. For the harmonized analysis, the time-to-event endpoint was derived from available information on progression, relapse, and death; information on secondary neoplasms was unavailable.
**APN311-202, V3**	EFS, OS	EFS, OS

EFS – event-free survival; OS – overall survival; PFS – progression-free survival

Supplementary Table S4. Comparison of patient characteristics and received treatment between RA and no-RA cohorts.

			No RA (N=36)	RA (N=44)	P value	Comment
**Age at DB initiation**		Median (IQR)	6.8 (4.7 to 8.1)	5.3 (3.8 to 7.8)	0.226	Included in the base-case PS model
		Mean (SD)	7.0 (3.4)	6.5 (4.0)		
**Age at diagnosis**		Median (IQR)	4.0 (2.9 to 5.5)	3.2 (2.3 to 5.3)	0.128	Not included in the base-case PS model
		Mean (SD)	4.5 (2.3)	4.2 (3.3)		
**Months since diagnosis**		Median (IQR)	25.0 (16.0 to 43.5)	18.6 (13.8 to 27.6)	0.107	Included in the base-case PS model
		Mean (SD)	35.8 (34.3)	27.9 (25.4)		
**Year of the diagnosis**		Median (IQR)	2014 (2012 to 2015)	2014 (2013 to 2014)	By design (0.252)	Not included in the base-case PS model
		Mean (SD)	2013.3 (3.1)	2013.0 (2.3)		
**Sex**	Male	n (%)	25 (69.4%)	25 (56.8%)	0.246	

			No RA (N=36)	RA (N=44)	P value	Comment
	Female		11 (30.6%)	19 (43.2%)		Included in the base-case PS model
**Ethnicity**	Asian	n (%)	0 (0%)	**2 (4.5%)**	0.401	Not included because fewer than three patients had the characteristic in at least one cohort
	Black		0 (0%)	**2 (4.5%)**		
	Italian/Philippines		0 (0%)	**1 (2.3%)**		
	not available		0 (0%)	**2 (4.5%)**		
	White or Caucasian		36 (100%)	36 (81.8%)		
	Cuban		0 (0%)	**1 (2.3%)**		
**Baseline performance score**	70	n (%)	1 (2.8%)	0 (0%)	**0.001** (0.198 without 'missing' level)	- (Not included in the PS model because 15/44 values were missing in the RA cohort and none were missing in the no-RA cohort; no imputation was performed)
	80		2 (5.6%)	0 (0%)		
	90		15 (41.7%)	8 (18.2%)		
	100		18 (50%)	21 (47.7%)		
	missing		0 (0%)	**15 (34.1%)**		
**Baseline MIBG scan**	soft tissue lesions	n (%)	12 (33.3%)	22 (50%)	0.134	Included in the base-case PS model
	distant lymph nodes	n (%)	**0 (0%)**	5 (11.4%)	**0.037**	Not included because fewer than three patients had the characteristic in at least one cohort
	liver lesions	n (%)	**2 (5.6%)**	5 (11.4%)	0.360	Not included because fewer than three patients had the characteristic in at least one cohort
	skeletal lesions	n (%)	27 (75%)	28 (63.6%)	0.275	Included in the base-case PS model
**Country**	Austria	n (%)	1 (2.8%)	3 (6.8%)	By design (**<0.001**)	Not included in the base-case PS model
	Belgium		0 (0%)	3 (6.8%)		
	Germany		35 (97.2%)	0 (0%)		
	Spain		0 (0%)	2 (4.5%)		

			No RA (N=36)	RA (N=44)	P value	Comment
	France		0 (0%)	3 (6.8%)		
	Georgia		0 (0%)	1 (2.3%)		
	Ireland		0 (0%)	1 (2.3%)		
	Israel		0 (0%)	3 (6.8%)		
	Italy		0 (0%)	13 (29.5%)		
	Poland		0 (0%)	4 (9.1%)		
	United Kingdom		0 (0%)	11 (25%)		
**INSS at diagnosis**	1	n (%)	2 (5.6%)	1 (2.3%)	0.342	Not included in the base-case PS model
	2A		0 (0%)	1 (2.3%)		
	2B		0 (0%)	2 (4.5%)		
	3		1 (2.8%)	3 (6.8%)		
	4		33 (91.7%)	35 (79.5%)		
	4s		0 (0%)	2 (4.5%)		
**INSS at diagnosis**	INSS=4	n (%)	33 (91.7%)	35 (79.5%)	0.131	Included in the base-case PS model
	other		3 (8.3%)	9 (20.5%)		
**Disease status**	Relapsed	n (%)	18 (50%)	14 (31.8%)	0.099	Included in the base-case PS model
	Refractory		18 (50%)	30 (68.2%)		
MYCN	Non-amplified	n (%)	30 (83.3%)	35 (79.5%)	0.418 (0.479 without 'missing' level)	Not included in the base-case PS model
	Amplified		5 (13.9%)	9 (20.5%)		
	missing		1 (2.8%)	0 (0%)		
MYCN	Other *MYCN*	n (%)	31 (86.1%)	35 (79.5%)	0.442	Dichotomized and included in the base-case PS model
	Amplified		5 (13.9%)	9 (20.5%)		
**Frontline treatment**	Chemotherapy	n (%)	34 (94.4%)	42 (95.5%)	0.837	Not included in the base-case PS model
	High-dose therapy	n (%)	27 (75%)	27 (61.4%)	0.195	Not included in the base-case PS model
	Local therapy	n (%)	34 (94.4%)	30 (68.2%)	**0.003**	Not included in the base-case PS model
	Maintenance therapy	n (%)	25 (69.4%)	8 (18.2%)	**<0.001**	Included in the base-case PS model

			No RA (N=36)	RA (N=44)	P value	Comment
	Radiotherapy	n (%)	13 (36.1%)	23 (52.3%)	0.148	Included in the base-case PS model
	Surgery	n (%)	32 (88.9%)	25 (56.8%)	**0.002**	Included in the base-case PS model
	Anti-GD2 maintenance	n (%)	3 (8.3%)	0 (0%)	0.051	Evaluated in a previously performed sensitivity analysis
	High-dose therapy: MIBG	n (%)	13 (36.1%)	8 (18.2%)	0.070	Included in the base-case PS model
	High-dose therapy: BuMEL	n (%)	12 (33.3%)	24 (54.5%)	0.058	Included in the base-case PS model
	High-dose therapy: CEM	n (%)	12 (33.3%)	**2 (4.5%)**	**0.001**	Not included because fewer than three patients had the characteristic in at least one cohort
	Chemotherapy: rapid COJEC	n (%)	5 (13.9%)	29 (65.9%)	**<0.001**	Included in the base-case PS model
	Chemotherapy: MSK m.N7 (5 cycles)	n (%)	**2 (5.6%)**	10 (22.7%)	**0.032**	Not included because fewer than three patients had the characteristic in at least one cohort
**Cycles with DB dose of at least 50 mg/m2**	1	n (%)	2 (5.6%)	2 (4.5%)	0.648	Evaluated in a previously performed sensitivity analysis based on the approach described in Reference 19
	2		4 (11.1%)	7 (15.9%)		
	3		1 (2.8%)	3 (6.8%)		
	4		3 (8.3%)	1 (2.3%)		
	5		26 (72.2%)	31 (70.5%)		
**Initiated DB treatment cycles [19]**	1	n (%)	2 (5.6%)	2 (4.5%)	0.928	Evaluated in a previously performed sensitivity analysis based on the
	2		4 (11.1%)	6 (13.6%)		
	3		1 (2.8%)	2 (4.5%)		
	4		2 (5.6%)	1 (2.3%)		
	5		27 (75%)	33 (75%)		

			No RA (N=36)	RA (N=44)	P value	Comment
						approach described in Reference 19
**Cumulative DB dose (mg/m2)**		Median (IQR)	466.1 (396.7 to 476.5)	474.6 (328.9 to 497.4)	0.178	Evaluated in a previously performed sensitivity analysis based on the approach described in Reference 19
		Mean (SD)	405.9 (121.6)	404.5 (127.2)		
**First subsequent anticancer therapy**	Local therapy	n (%)	1 (2.8%)	4 (9.1%)	**0.004**	Evaluated in a previously performed sensitivity analysis
	Retinoid / transplant consolidation		4 (11.1%)	1 (2.3%)		
	Targeted / biologic systemic therapy		0 (0%)	3 (6.8%)		
	Cytotoxic chemotherapy		0 (0%)	9 (20.5%)		
	Missing / not applicable		31 (86.1%)	27 (61.4%)		

BuMEL – busulfan and melphalan; CEM – carboplatin, etoposide, melphalan; COJEC – cisplatin, vincristine, carboplatin, etoposide, cyclophosphamide; DB – dinutuximab beta; IQR – interquartile range; SD – standard deviation; INSS – International Neuroblastoma Staging System; MIBG – metaiodobenzylguanidine; *MYCN* – *MYCN* oncogene; MSK m.N7 – Memorial Sloan Kettering modified N7 protocol; RA – isotretinoin (13-cis-retinoic acid); SA – sensitivity analysis

Supplementary Table S5. Scenarios of the sensitivity analyses.

Type of the analysis	SA description
EFS and OS from the 1st DB administration, no weighting	Naïve comparison without weighting
Post-landmark EFS and OS with DB exposure adjustment	The final weight = the product of IPSW and PS overlap weights; exposure metric: cycles with DB initiation
EFS and OS from the 1st DB administration, PS weighted	Study 304: 4 patients with known previous anti-GD2 treatment were excluded
EFS and OS from the 1st DB administration, PS weighted	Exploratory analysis excluding eight patients who received selected post-baseline anticancer therapies, including retinoid/transplant consolidation or targeted/biologic systemic therapy. Because the exclusion criterion was based on subsequent treatment, this analysis may be affected by selection bias and was not interpreted as adjustment for baseline confounding
EFS and OS from the 1st DB administration, PS weighted	Doubly robust Cox model

Type of the analysis	SA description
Post-landmark EFS and OS with DB exposure adjustment	Exposure metric: cycles with DB dose of at least 50 mg/m^2; the final weight = the product of IPSW and PS overlap weights
Post-landmark EFS and OS with DB exposure adjustment	Exposure metric: cumulative dose (mg/m^2); the final weight = the product of IPSW and PS overlap weights
Post-landmark EFS and OS with DB exposure adjustment	The final weight = PS overlap weights from the IPSW-weighted model; exposure metric: cycles with DB initiation
Post-landmark EFS and OS with DB exposure adjustment	The final weight = the product of IPSW and PS overlap weights, but IPSWs were estimated without the cohort indicator; exposure metric: cycles with DB initiation
Post-landmark EFS and OS with DB exposure adjustment	The final weight = the PS overlap weights (population: those without event at EOT); exposure metric: cycles with DB initiation

DB – dinutuximab beta; EFS – event-free survival; EOT – end of treatment; IPSW – inverse probability of selection weights; OS – overall survival; PS – propensity score; SA – sensitivity analysis. No sensitivity analysis involving deterministic assignment, complete-case restriction, or model-based imputation of baseline performance status was performed because all missing values occurred in one source-study cohort and no validated information was available to support the required assumptions.

Supplementary Table S6. Results of the primary analyses and other sensitivity analyses.

<table>
<tr><th>Analysis scenario</th><th>Kaplan-Meier plots</th><th>Cox models</th></tr>
<tr>
<td>Primary base-case analysis, presented for reference</td>
<td>
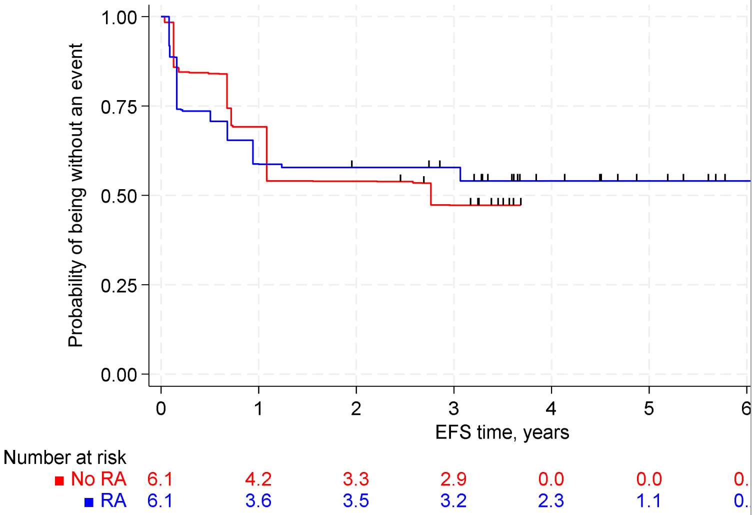

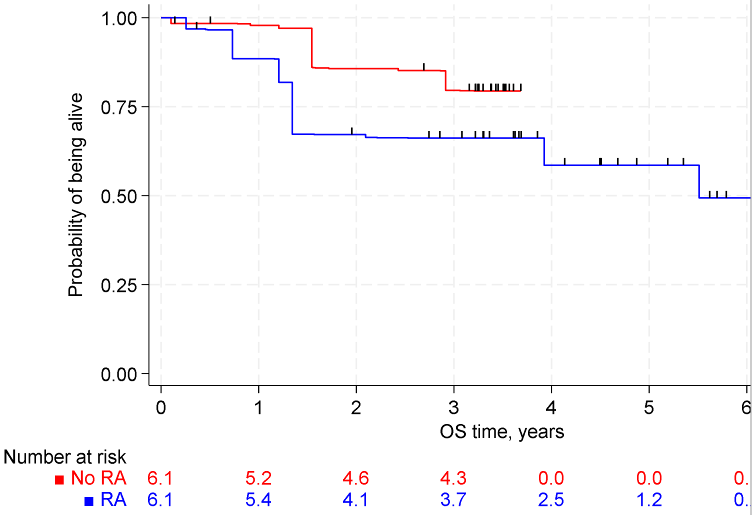

</td>
<td>
<table>
<tr><th>RA vs no-RA</th><th>HR (95% CI)</th><th>p Value</th></tr>
<tr><td>EFS</td><td>0.92 (0.31 to 2.73)</td><td>0.884</td></tr>
<tr><td>OS</td><td>1.95 (0.46 to 8.20)</td><td>0.364</td></tr>
</table>
</td>
</tr>
</table>

<table>
<tr><th>Analysis scenario</th><th>Kaplan-Meier plots</th><th>Cox models</th></tr>
<tr>
<td>Naïve comparison</td>
<td>
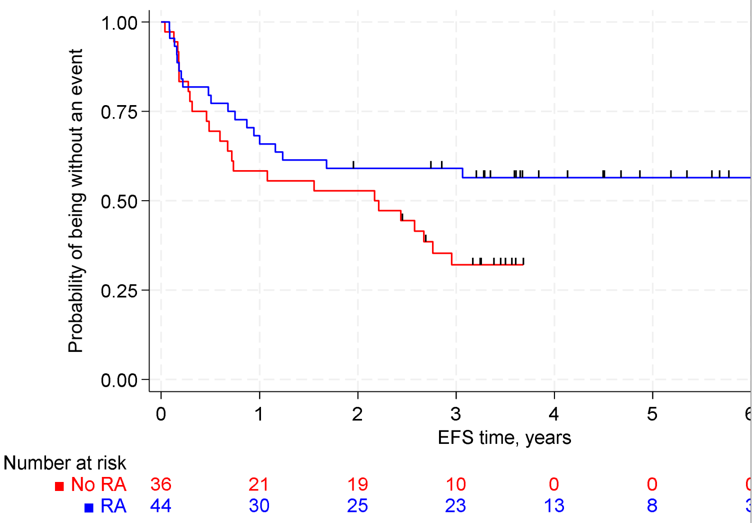

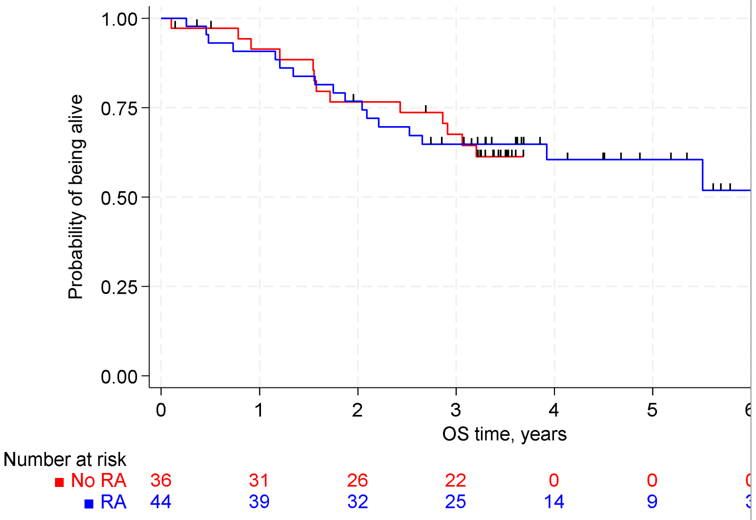

</td>
<td>
<table>
<tr><th>RA vs no-RA</th><th>HR (95% CI)</th><th>p Value</th></tr>
<tr><td>EFS</td><td>0.57 (0.31 to 1.04)</td><td>0.067</td></tr>
<tr><td>OS</td><td>0.96 (0.46 to 1.99)</td><td>0.903</td></tr>
</table>
</td>
</tr>
</table>

<table>
<tr><th>Analysis scenario</th><th>Kaplan-Meier plots</th><th>Cox models</th></tr>
<tr>
<td>Primary exploratory post-EOT landmark analysis with DB exposure adjustment; final weight = IPSW × PS overlap weights; exposure metric = initiated DB cycles</td>
<td>
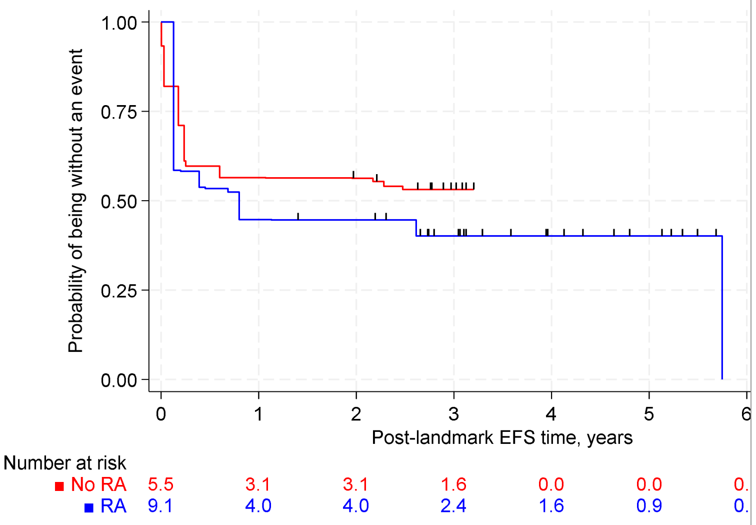

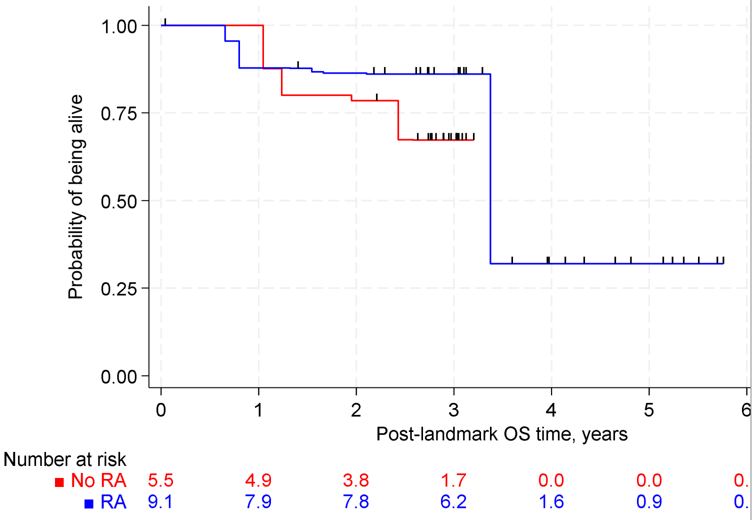

</td>
<td>
<table>
<tr><th>RA vs no-RA</th><th>HR (95% CI)</th><th>p Value</th></tr>
<tr><td>EFS</td><td>1.24 (0.37 to 4.12)</td><td>0.725</td></tr>
<tr><td>OS</td><td>0.44 (0.07 to 2.71)</td><td>0.374</td></tr>
</table>
</td>
</tr>
</table>

Analysis scenario	Kaplan-Meier plots	Cox models
**EFS and OS from the 1st DB administration, PS weighting + 4 patients with previous anti-GD2 treatment (Study 304) were excluded**	Kaplan-Meier plots below	Cox models below

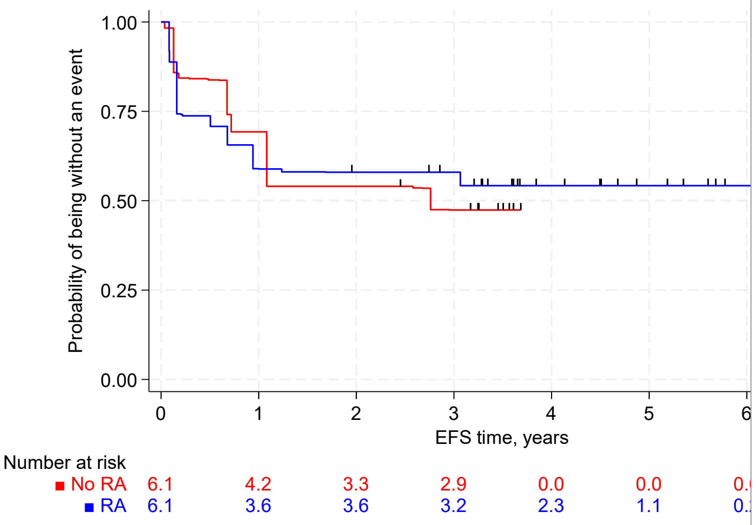

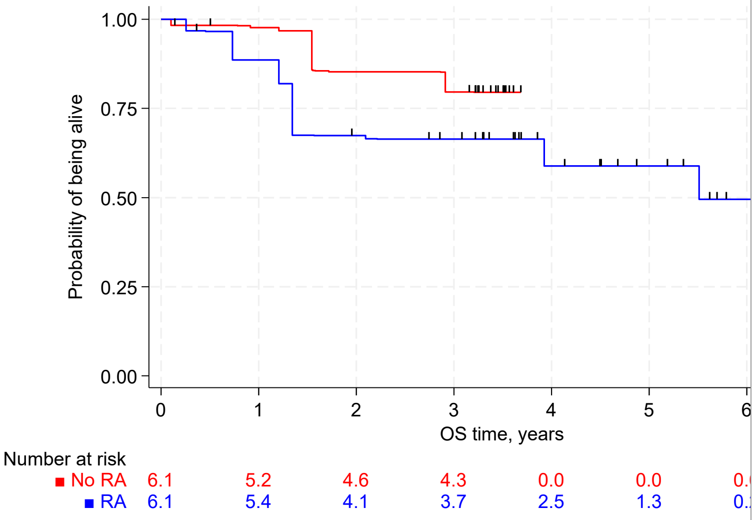

RA vs no-RA	HR (95% CI)	p Value
**EFS**	0.92 (0.31 to 2.72)	0.878
**OS**	1.93 (0.46 to 8.20)	0.371

<table>
<tr><th>Analysis scenario</th><th>Kaplan-Meier plots</th><th>Cox models</th></tr>
<tr>
<td>Previously performed exploratory analysis excluding eight patients who received selected post-baseline therapies; susceptible to selection bias and not interpreted as adjustment for baseline confounding</td>
<td>

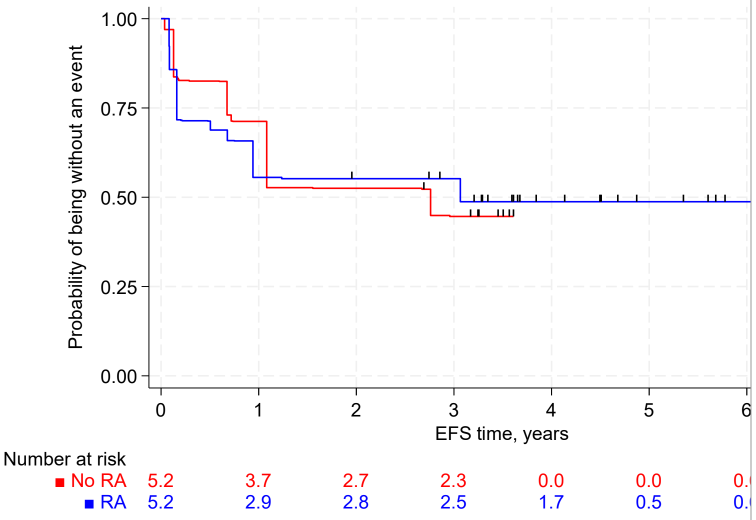

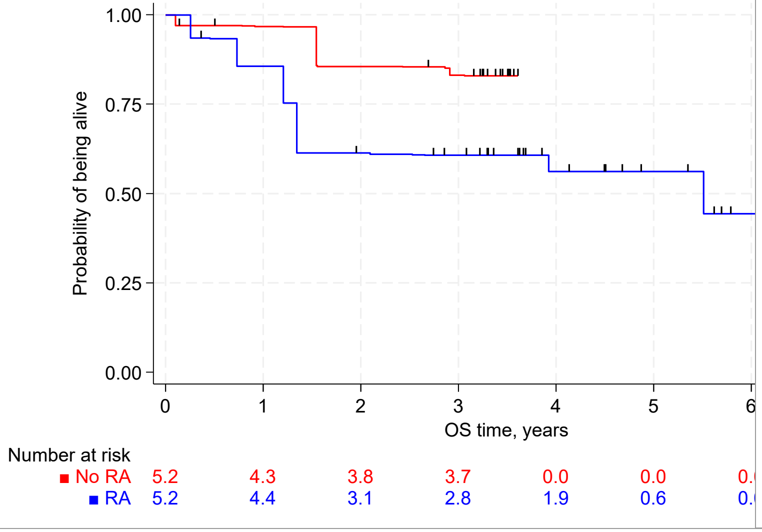

</td>
<td>

RA vs no-RA	HR (95% CI)	p Value
**EFS**	0.99 (0.33 to 2.95)	0.985
**OS**	2.75 (0.60 to 12.63)	0.195

</td>
</tr>
</table>

<table>
<tr><th>Analysis scenario</th><th>Kaplan-Meier plots</th><th>Cox models</th></tr>
<tr>
<td>EFS and OS from the 1st DB administration, PS weighting
+
doubly robust Cox models</td>
<td>
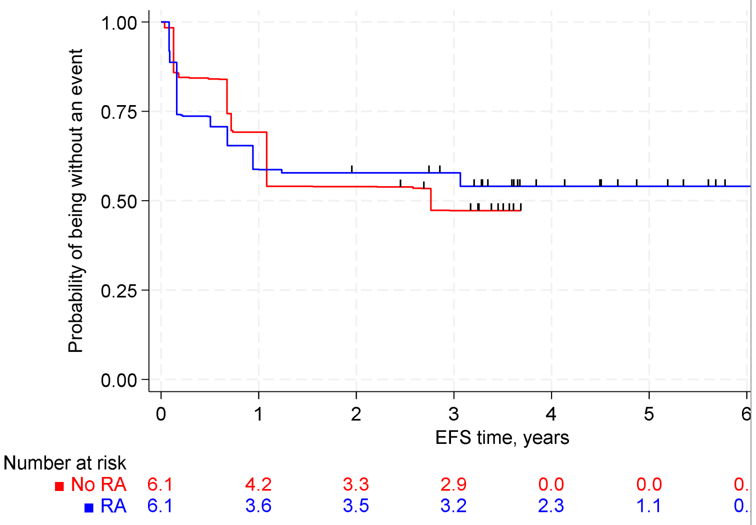

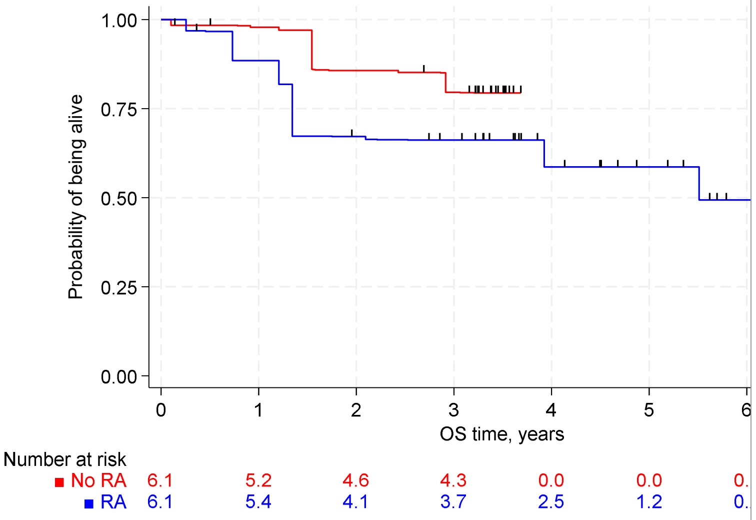

</td>
<td>
<table>
<tr><th>RA vs no-RA</th><th>HR (95% CI)</th><th>p Value</th></tr>
<tr><td>EFS</td><td>0.98 (0.16 to 5.79)</td><td>0.979</td></tr>
<tr><td>OS</td><td>3.62 (0.06 to 220.90)</td><td>0.539</td></tr>
</table>
</td>
</tr>
</table>

Analysis scenario	Kaplan-Meier plots	Cox models

Analysis scenario

Post-landmark EFS and OS with DB exposure adjustment
+
Exposure metric: cycles with DB dose of at least 50 mg/m^2

Kaplan-Meier plots

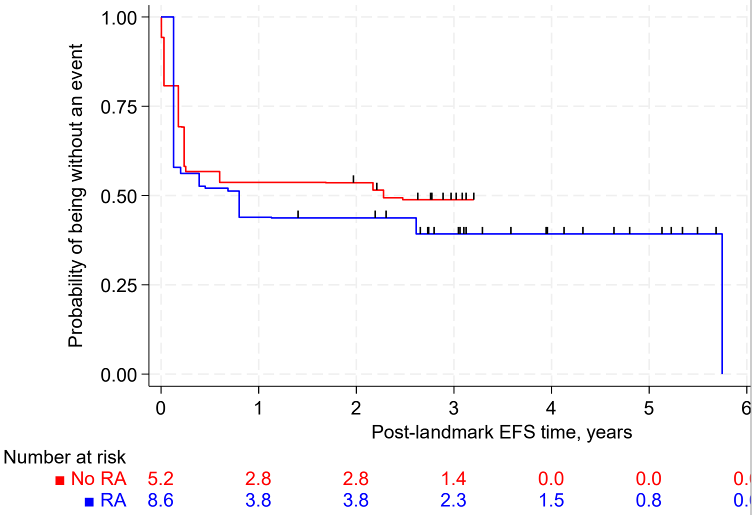

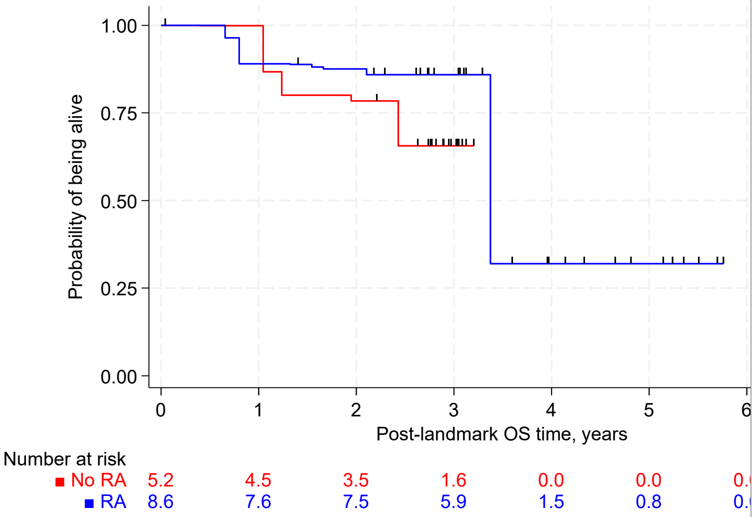

Cox models

RA vs no-RA	HR (95% CI)	p Value
**EFS**	1.14 (0.35 to 3.77)	0.827
**OS**	0.42 (0.07 to 2.47)	0.334

Analysis scenario	Kaplan-Meier plots	Cox models
**Post-landmark EFS and OS with DB exposure adjustment + Exposure metric: cumulative dose (mg/m^2)**	See plots below	See table below

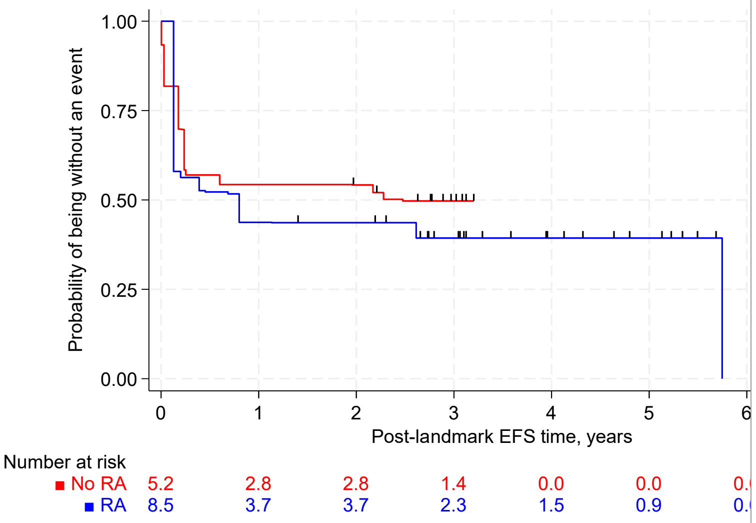

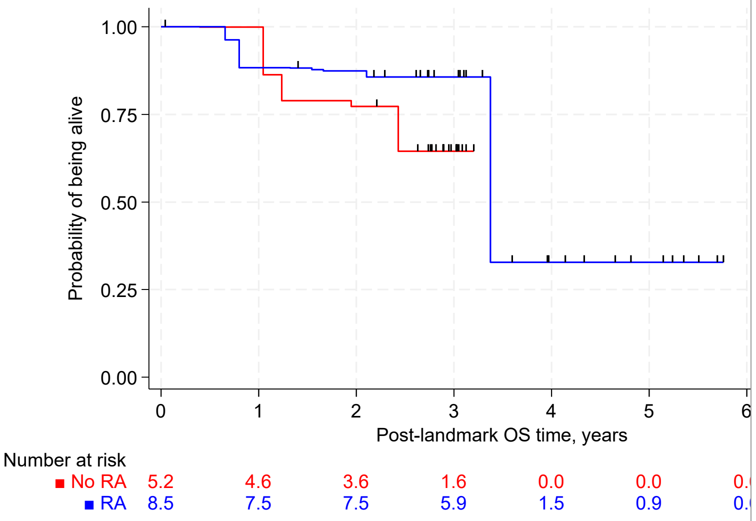

RA vs no-RA	HR (95% CI)	p Value
**EFS**	1.17 (0.36 to 3.87)	0.793
**OS**	0.41 (0.07 to 2.49)	0.331

<table>
<tr><th>Analysis scenario</th><th>Kaplan-Meier plots</th><th>Cox models</th></tr>
<tr>
<td>Post-landmark EFS and OS with DB exposure adjustment
+
The final weight = PS overlap weights from the IPSW-weighted model</td>
<td>
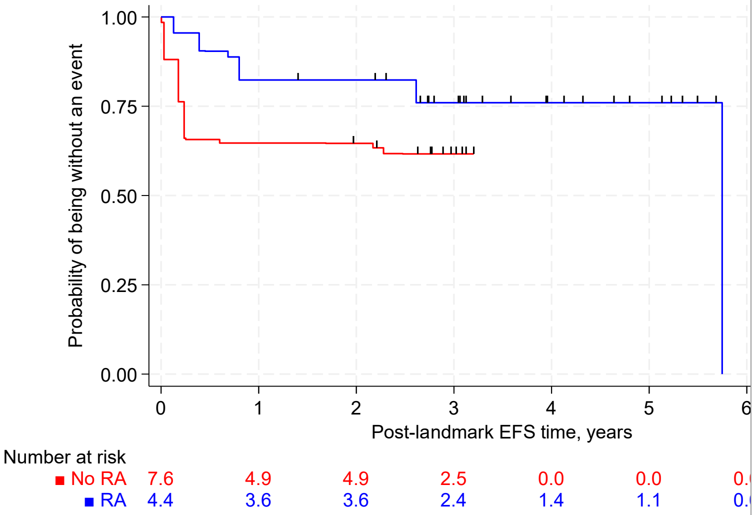

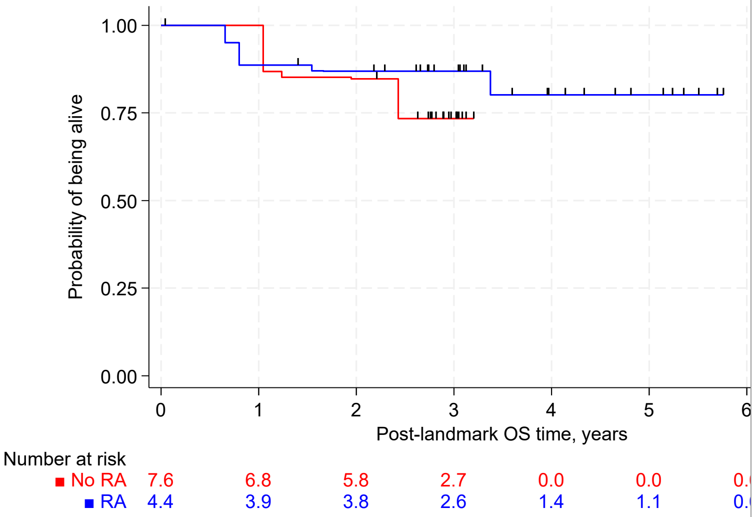

</td>
<td>
<table>
<tr><th>RA vs no-RA</th><th>HR (95% CI)</th><th>p Value</th></tr>
<tr><td>EFS</td><td>0.51 (0.13 to 1.99)</td><td>0.334</td></tr>
<tr><td>OS</td><td>0.54 (0.09 to 3.20)</td><td>0.502</td></tr>
</table>
</td>
</tr>
</table>

<table>
<tr><th>Analysis scenario</th><th>Kaplan-Meier plots</th><th>Cox models</th></tr>
<tr>
<td>Post-landmark EFS and OS with DB exposure adjustment
+
The final weight = the product of IPSW and PS overlap weights, but IPSWs were estimated without the cohort indicator</td>
<td>
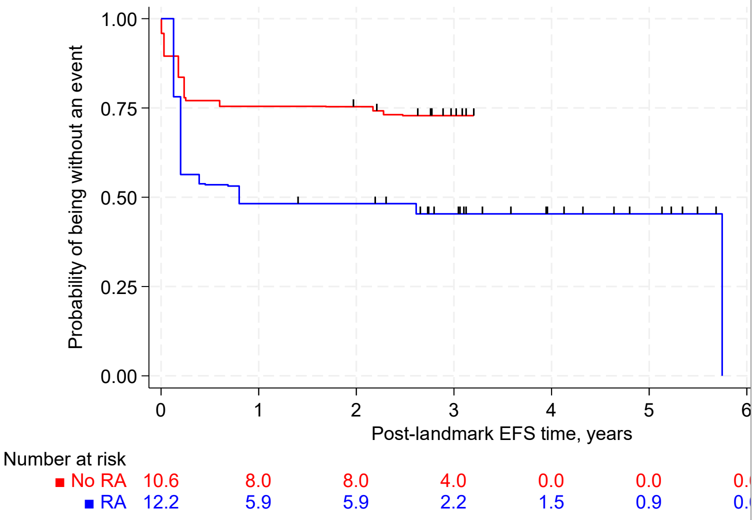

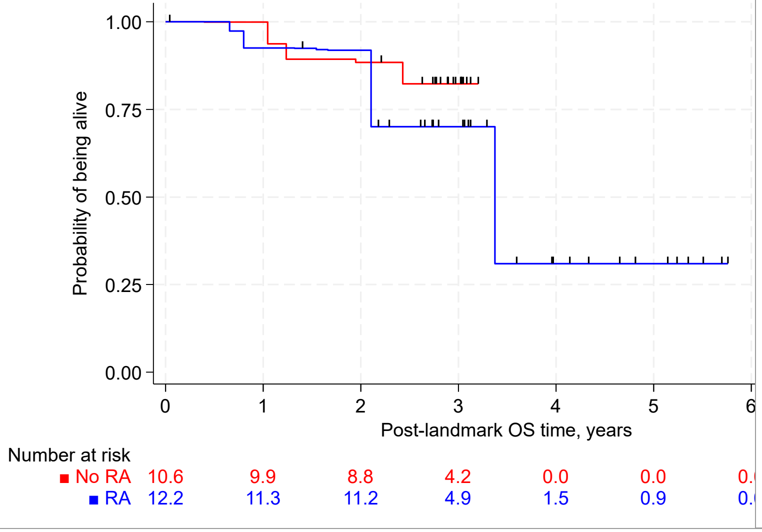

</td>
<td>
<table>
<tr><th>RA vs no-RA</th><th>HR (95% CI)</th><th>p Value</th></tr>
<tr><td>EFS</td><td>2.22 (0.51 to 9.69)</td><td>0.290</td></tr>
<tr><td>OS</td><td>1.74 (0.29 to 10.44)</td><td>0.543</td></tr>
</table>
</td>
</tr>
</table>

<table>
<tr><th>Analysis scenario</th><th>Kaplan-Meier plots</th><th>Cox models</th></tr>
<tr>
<td>Post-landmark EFS and OS with DB exposure adjustment
+
The final weight = the PS overlap weights (population = those without event at EOT)</td>
<td>
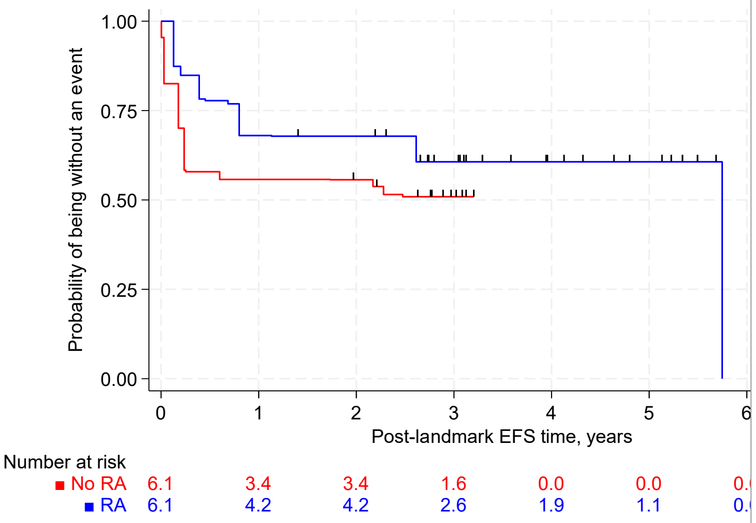

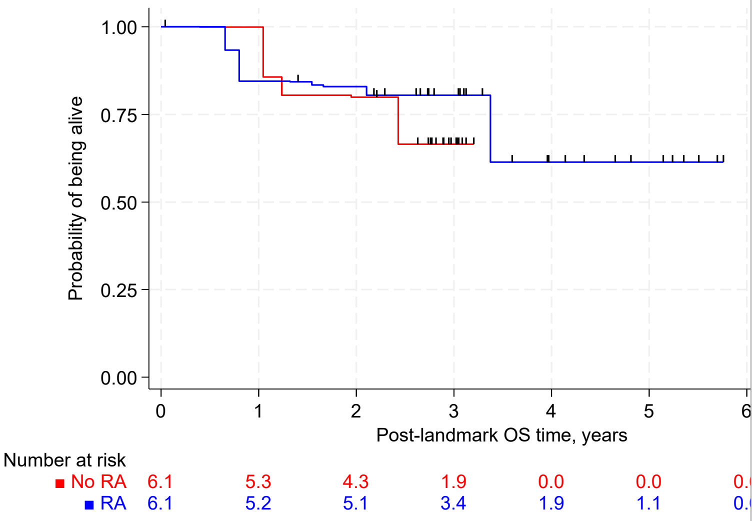

</td>
<td>
<table>
<tr><th>RA vs no-RA</th><th>HR (95% CI)</th><th>p Value</th></tr>
<tr><td>EFS</td><td>0.66 (0.20 to 2.20)</td><td>0.497</td></tr>
<tr><td>OS</td><td>0.63 (0.13 to 3.02)</td><td>0.564</td></tr>
</table>
</td>
</tr>
</table>

All estimates presented in this table were available from the previously validated analytical output. No new sensitivity analyses, model variants, missing-data reconstructions, or subgroup analyses were performed during the revision. The reported P values are descriptive and should not be interpreted as evidence of equivalence, absence of effect, or covariate balance. Numbers at risk in weighted Kaplan–Meier plots are weighted sums, not patient counts or effective sample sizes; the unweighted naïve analysis displays patient counts.
